# A stoichiometric terbium-europium dyad molecular thermometer: energy transfer properties

**DOI:** 10.1038/s41377-018-0097-7

**Published:** 2018-11-28

**Authors:** Guochen Bao, Ka-Leung Wong, Dayong Jin, Peter A. Tanner

**Affiliations:** 1 Department of Chemistry, Hong Kong Baptist University, Kowloon Tong, Hong Kong SAR People’s Republic of China; 2 Institute for Biomedical Materials and Devices (IBMD), Faculty of Science, University of Technology Sydney, Sydney, NSW 2007 Australia; 3 School of Mathematical and Physical Sciences, Faculty of Science, University of Technology Sydney, Sydney, NSW 2007 Australia

## Abstract

The optical thermometer has shown great promise for use in the fields of aeronautical engineering, environmental monitoring and medical diagnosis. Self-referencing lanthanide thermo-probes distinguish themselves because of their accuracy, calibration, photostability, and temporal dimension of signal. However, the use of conventional lanthanide-doped materials is limited by their poor reproducibility, random distance between energy transfer pairs and interference by energy migration, thereby restricting their utility. Herein, a strategy for synthesizing hetero-dinuclear complexes that comprise chemically similar lanthanides is introduced in which a pair of thermosensitive dinuclear complexes, cycTb-phEu and cycEu-phTb, were synthesized. Their structures were geometrically optimized with an internuclear distance of approximately 10.6Å. The sensitive linear temperature-dependent luminescent intensity ratios of europium and terbium emission over a wide temperature range (50–298K and 10–200K, respectively) and their temporal dimension responses indicate that both dinuclear complexes can act as excellent self-referencing thermometers. The energy transfer from Tb^3+^ to Eu^3+^ is thermally activated, with the most important pathway involving the ^7^F_1_ Eu^3+^ *J*-multiplet at room temperature. The energy transfer from the antenna to Eu^3+^ was simulated, and it was found that the most important ligand contributions to the rate come from transfers to the Eu^3+^ upper states rather than direct ligand–metal transfer to ^5^D_1_ or ^5^D_0_. As the first molecular-based thermometer with clear validation of the metal ratio and a fixed distance between the metal pairs, these dinuclear complexes can be used as new materials for temperature sensing and can provide a new platform for understanding the energy transfer between lanthanide ions.

## Luminescent lanthanides for nano-scale optical thermometers

A metal-organic complex developed by researchers in China and Australia could greatly improve the accuracy of molecular-scale luminescent thermometers. The ability to measure temperatures in nano-sized spaces, such as within the human body or on microchips, holds great appeal. This promising approach uses molecular frameworks containing lanthanide ions, which display luminescence that is strongly dependent on their temperature. Ka-Leung Wong at Hong Kong Baptist University and co-workers synthesized two complexes containing a single pair of terbium and europium. By smart design and architecting at molecular level, the team fixed two ions with different emission colors at a distance of 1.06 nm, enabling effective energy transfer between the two single emitters. The luminescence from each ion responds differently as the temperature changes. Hence accurate temperature measurement is achieved via comparison of the luminescence from each emitter.

## Introduction

Luminescent physical sensors for monitoring temperature have shown great promise for use in the fields of aeronautical engineering, environmental engineering, and industrial processes^1–4^. They have distinguished advantages over traditional thermometers in terms of a fast response, a high sensitivity, and a tolerance to extreme atmospheres^5–7^. In particular, self-referencing optical thermometers do not require additional calibration of the emission intensity and are more accurate due to noninvasive operation^8,9^.

Materials containing two different lanthanide ions are attractive for the construction of a self-referencing thermometer^10^. Lanthanide luminescent materials have sharp emission bands^11,12^ and a large energy shift between the antenna absorption and lanthanide emission^13^, which distinguishes them from other luminescent materials, such as organic dyes or quantum dots^14^. In addition to these properties, lanthanide luminescence has a long lifetime, allowing time-gated techniques to increase the signal-to-noise ratio^15^. The temperature-dependent quenching and energy transfer between the dopant ions or between the host and lanthanide ion allow selective luminescent responses for different lanthanide ions with a change in temperature. Many lanthanide materials have been developed for temperature monitoring, such as lanthanide-doped inorganic nanocrystals^16^, metal-organic frameworks (MOFs)^5,17^, polymers^1^, and co-doped complexes^18–20^. The reported maximum temperature sensitivities of recent systems involving energy transfer between two different lanthanide ions for ratiometric emission measurements are collected in Table S13. The stoichiometry of these systems is a critical parameter, and the sensitivities usually vary widely with temperature.

However, it is difficult to obtain exactly the same concentrations and qualities from different prepared batches of doped materials; the distance between the donor ion and acceptor ion, which is a crucial parameter for energy transfer, is random and can only be estimated by statistical treatment, and a mixed system with these materials allows donor–donor and acceptor–acceptor energy migration, making the kinetics of transfer more complicated. Hence, the hetero-dinuclear lanthanide complex, with its fixed distance between donor and acceptor^12^, its definite structure and stoichiometric ratio of donor and acceptor, has great potential for development as a luminescent self-referencing thermosensitive indicator. The donor and acceptor in the dinuclear complex are fixed at a certain distance so that the pairwise energy transfer rates can be obtained without statistical treatment. With a stoichiometric arrangement of different lanthanide ions at each binding center, the intramolecular energy transfer between the ions is scarcely interfered with by donor–donor and acceptor–acceptor energy migration. The antenna effect of the chelating chromophore, which possesses a large absorption cross section, allows more incident light to be absorbed and transferred to a lanthanide ion, making such complexes brighter than most lanthanide-doped inorganic nanocrystals. Unsurprisingly, few literature reports^18,21^ include an investigation of the energy transfer processes using lanthanide hetero-dinuclear complexes - and even then with the co-presence of homo-dinuclear species in the complex mixture. Even though great effort has been expended to synthesize pure hetero-polynuclear complexes^22,23^ with different chemically similar lanthanide ions, there remains a considerable need for a simple strategy for controlling the formation of hetero-dinuclear complexes. This is a severe challenge for lanthanide chemists.

**Fig. 1:**
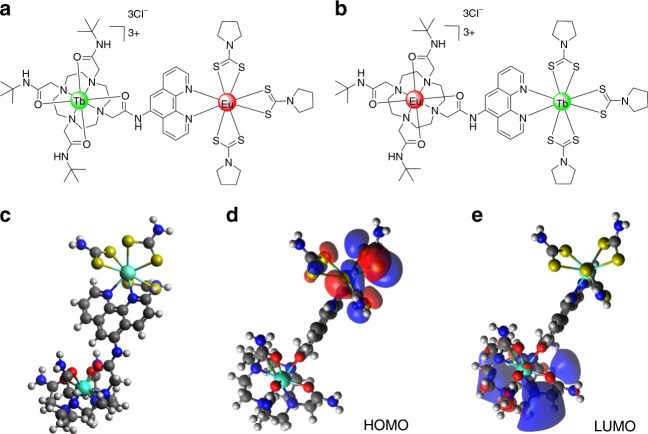
**The structures of complexes.** **a** cycTb-phEu and **b** cycEu-phTb, and **c** optimized geometry of the simplified 100-atom structure of cycTb-phEu as determined using the PBeh-3c functional^24^ with basis def2-SVP^25^ and effective core potentials for Tb and Eu^26^ in ORCA^27^ (refer to the SI for details). The charge-transfer nature of the **d** HOMO and **e** LUMO are shown

Herein, we report a pair of hetero-dinuclear lanthanide complexes, cycTb-phEu and cycEu-phTb, as molecular-based luminescent temperature sensors. In order to avoid mixing the two chemically similar lanthanide ions, europium and terbium, they are situated in two distinct binding sites using two different synthesis steps. The clear validation of the metal ratio and the fixed distance between the energy donor and acceptor make the complexes outweigh other materials, such as doped crystals, MOFs and polymers, both in terms of the understanding energy transfer and in temperature sensing performance. The 1,10-phenanthroline (phen) moiety serves as the chromophore to sensitize europium and terbium, giving an increased temperature-dependent luminescence emission ratio for europium over terbium. The luminescence from terbium and from europium is reduced in intensity with different rates as the temperature increases, and it involves the process of energy transfer from the chromophore to each ion and that from terbium to europium. Complexes cycTb-phEu and cycEu-phTb (Fig. 1) exhibit remarkably different photophysics due to the uniqueness of the two metal binding centers, and their energy transfer processes have been studied in detail.

## Results

### Structure

Refer to Fig. S1 for the structures and names of the complexes. The 166-atom structure of cycTb-phEu was modeled by MOPAC^28,29^ using the semi-empirical RM1 software in the LUMPAC program^30,31^, and the structure is shown in Fig. S13. Both Tb and Eu are 8-coordinated. Eu is coordinated to six S and to two N, whereas Tb is coordinated to four O and four N. A simplified 100-atom structure with aliphatic rings replaced by CH_2_ groups was employed for the optimization using the PBeh-3c functional in ORCA^24–27^ (Fig. 1). A comparison of the bond distances for these two different optimizations is provided in Table S10.

### Synthesis

Scheme S1 shows the synthesis of the ligands and complexes. Ligand **2** (cyclen 1,10-phenanthroline) was prepared by a substitution reaction of 2-bromoacetyl bromide with 1,10-phenanthrolin-5-amine in the presence of K_2_CO_3_ in DCM for 22 h to give **1**, which was followed by coupling with a triarmed cyclen in MeCN in the presence of NaHCO_3_ at room temperature, giving **2** in a 95.8% yield. The triarmed cyclen **1** was prepared via two-step substitution reactions according to the literature^13^. Complexes **3** (cycLn^1^-phen) were formed by coordinating lanthanide chlorides with one equivalent of ligand **2** in a mixed solution of MeOH and H_2_O at room temperature, followed by precipitation with diethyl ether from methanol. Taking advantage of fast coordination to Ln(III) ions and less interference with the energy transfer from the 1,10-phenanthroline chromophore to the Ln(III) ions, pyrrolidine dithiocarbamate was chosen as the ligand for coordination with the second lanthanide ion. The synthesis of complexes **4** (cycLn^1^-phLn^2^) was achieved by coordinating lanthanide chloride with one equivalent of complex **3** and three equivalents of ammonium pyrrolidine-1-carbodithioate at room temperature, followed by precipitation with diethyl ether from methanol. The proton nuclear magnetic resonance (^1^H NMR) spectra of the La motif structure showed that three pyrrolidine dithiocarbamates were coordinated to the complex, which is consistent with a previous report^32^. This synthetic strategy provides a platform to obtain hetero-dinuclear complexes that consist of chemically similar lanthanide ions, one of which is not present in both ligand structures.

### Singlet and triplet states

**Fig. 2:**
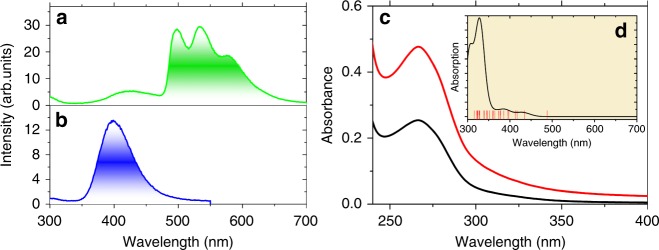
**Optical spectra.** The 77 K phosphorescence **a** and 298 K fluorescence **b** of cycLa-phLa (*λ*_exc_ = 273 nm) in the solid state. **c** Room temperature absorption spectrum of cycLa-phEu in buffer solution at pH 7.4 at two concentrations: black, 5 μM; red, 10 μM. The insert **d** shows the simulated absorption spectrum in the gas phase between 300 and 700 nm as determined from LUMPAC^30^ with the triplet levels calculated to low energy of 316 nm marked by vertical lines. The singlet transitions were broadened with widths of 2000 cm^−1^

The singlet and triplet energy levels were experimentally studied by using the La motif complex, cycLa-phLa, since La^3+^ does not exhibit 4f-4f electron transitions. The large La^3+^ ion generates a heavy atom effect towards the surrounding organic ligand and induces spin-orbit coupling, which accommodates intersystem crossing from singlets to triplets. Thus, phosphorescence occurs. The fluorescence from the solid La^3+^ complex was measured at room temperature, while the phosphorescence was recorded at 77 K (Fig. 2). The room temperature emission under broad band 273 nm excitation was observed for cycLa-phLa with a maximum at 398 nm (25121 cm^−1^) (Fig. 2b), which is attributed to a π-π* singlet transition of the ligand. At lower temperature, the fluorescence intensity decreases, the band redshifts, and phosphorescence from T_1_ dominates at longer wavelengths (Fig. 2a). The zero phonon line of the T_1_ → S_0_ transition is at 497 nm (20,124 cm^−1^), and there is a progression of the totally symmetric ring C = N mode at 1471 ± 6 cm^−1^ to lower energy (compare with the Fourier transform infrared (FT-IR) spectrum, Fig. S2). The excitation spectrum of the phosphorescence (Fig. S3a) demonstrates the population of T_1_ from a singlet state at ~400 nm. The excitation spectrum of the singlet fluorescence (Fig. S3b) shows a further singlet state at 273 nm. A strong band at or near this wavelength is observed for all of the cycLn-phLn systems in solution, for example, cycLa-phEu (Fig. 2c). In fact, the room temperature excitation spectra of cycEu-phLa (Fig. S4) and cycLa-phEu (Fig. S5) demonstrate the participation of other singlet states in the energy transfer routes from the ligand to Eu^3+^. Notably, by comparing the intensity of the ligand and Eu^3+^ absorption bands in the excitation spectra, the ligand–metal energy transfer is more efficient when Eu^3+^ is bound to the phen rather than to the cyclen moiety (Figs. S4 and S5).

The triplet lifetime was determined to be 0.26 s at 77 K (Fig. S6a), whereas the singlet lifetime was measured at room temperature to be 1.6 ns (Fig. S6b).

### Lanthanide luminescence at low temperature

**Fig. 3:**
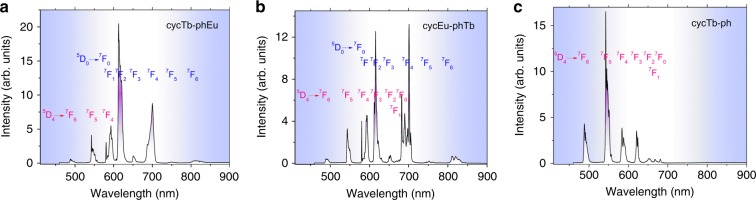
**The emission spectra (*λ***_**exc**_** = 355 nm) of complexes.** cycTb-phEu **a**, cycEu-phTb **b**, and cycTb-phen **c** at 10 K in the solid state. The Tb^3+^ transitions are marked in red, and those of Eu^3+^ are marked in blue. Refer to Tables S1 and S2. The relative intensities are not to scale for **a**–**c**

The emission spectra of complexes cycTb-phEu and cycEu-phTb were measured in the solid state at 10 K with excitation at 355 nm into the ligand absorption band (Fig. 3). These emission spectra exhibit the characteristic Eu^3+^ and Tb^3+^ emission bands: ^5^D_0_ → ^7^F_*J*_ (*J* = 0–6) for Eu^3+^ and ^5^D_4_ → ^7^F_*J*_ (*J* = 6–4) for Tb^3+^. In each case, the emission from Eu^3+^ dominates with the forced electric dipole transitions ^5^D_0_ → ^7^F_*J*_ (*J* *=* 2, 4) being the strongest and more intense than the magnetic dipole-allowed transition ^5^D_0_ → ^7^F_1_. The ^5^D_0_ → ^7^F_0_ transition, which is active for Eu^3+^ situated at sites with *C*_n_, *C*_nv_, and *C*_s_ symmetry, was observed as a single sharp band in each spectrum, indicating that only one type of complex is present. The spectral bands are listed in Tables S1 and S2 together with the derived Eu^3+^ and Tb^3+^ energy level data. For Eu^3+^, the major difference between the two spectra is the stronger relative intensity and number of resolved bands of the ^5^D_0_ → ^7^F_4_ transition for cycEu-phTb. Interestingly, the emission bands from either Eu^3+^ or Tb^3+^ situated in the more rigid cyclen binding site are sharper than when Eu^3+^ or Tb^3+^ is coordinated in the phenLn(pdtc)_3_-binding site in both dinuclear complexes.

The hypersensitive transition ^5^D_0_ → ^7^F_2_ is more influenced by the coordinative environment than other forced ED transitions and serves as an indicator of the local symmetry of a coordination site^33^. The area ratio of the ^5^D_0_ → ^7^F_2_ transition to that of ^5^D_0_ → ^7^F_1_ is 3.93 for cycTb-phEu and 2.33 for cycEu-phTb. The forced electric dipole transition, ^5^D_0_ → ^7^F_6_, exhibits a weak emission intensity in the luminescence spectrum of each binuclear complex.

The 10K emission spectrum of cycTb-phen is displayed in Fig. 3c. The near-environment of Tb^3+^ is the same as in cycTb-phEu (Fig. 3a); however, the (pdtc)_3_Eu moiety is absent. The Tb^3+^-Eu^3+^ distance in cycTb-phEu is 10.6 ± 0.1 Å. The crystal field levels of Tb^3+^, as deduced in Table S1 from the Tb^3+^ emission spectra in Fig. 3a, c, are the same within experimental error for cycTb-phen and cycTb-phEu. For example, the splittings of the *J*-multiplets ^7^F_6_ and ^7^F_5_ are 407 ± 5 cm^−1^ and 494 cm^−1^, respectively, in each case. This gives an indication of the “spectroscopic vision” of Tb^3+^ and shows the atomic core-like nature of 4f orbitals so that the crystal field experienced by Tb^3+^ is effectively the same for both systems.

### Emission decay lifetimes of Tb^3+^ and Eu^3+^ and temperature dependence

**Fig. 4:**
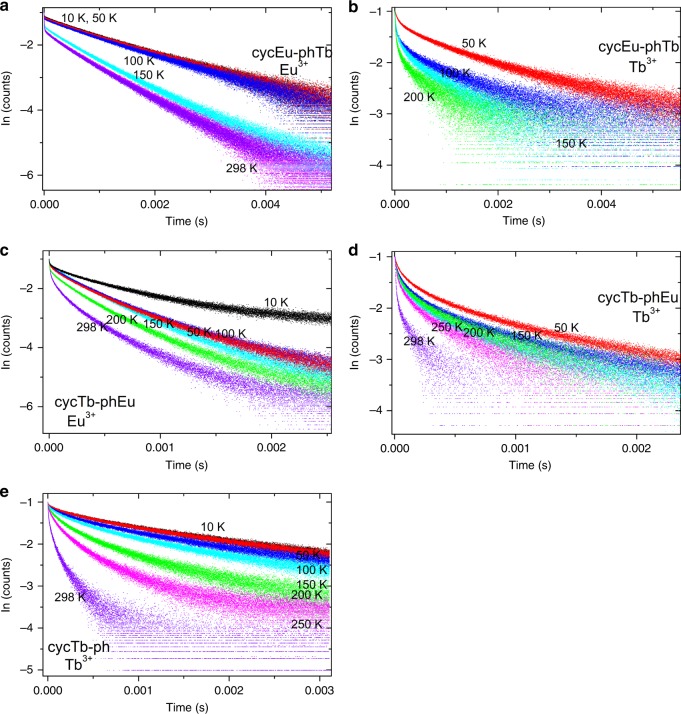
^**5**^**D**_**0**_** → **^**7**^**F**_**4**_ **Eu**^**3+**^ **and** ^**5**^**D**_**4**_ **→** ^**7**^**F**_**5**_ **Tb**^**3+**^ **emission decays (*λ***_**exc**_ **= 355 nm) at different temperatures.** cycEu-phTb: **a** Eu^3+^ and **b** Tb^3+^; cycTb-phEu **c** Eu^3+^ and **d** Tb^3+^; cycTb-phen **e** Tb^3+^. Black, 10 K; red, 50 K; blue, 100 K; cyan, 150 K; green, 200 K; magenta, 250 K; and violet, 298 K

The intensity of emission was monitored as a function of time after pulsed excitation for the ^5^D_0_ (Eu^3+^) and ^5^D_4_ (Tb^3+^) states by measuring the emission decays for the ^5^D_0_ → ^7^F_4_ transition of Eu^3+^ and for the ^5^D_4_ → ^7^F_5_ transition of Tb^3+^ because the spectral bands of these transitions are well-separated from those of other transitions. The excitation wavelength, 355 nm, overwhelmingly excites the phen antenna, which may then transfer energy to Eu^3+^ and Tb^3+^. A further energy transfer from Tb^3+^ to Eu^3+^ is well-documented in the literature;^34–40^ however, the use of excitation at 490 nm produced very weak emission in the present study due to weak absorption. Thus, we were unable to *directly* study this energy transfer process using this excitation wavelength. Fig. 4a–d display the Eu^3+^ ^5^D_0_ and Tb^3+^ ^5^D_4_ emission decays for cycTb-phEu and cycEu-phTb at various temperatures, whereas Fig. 4e shows the Tb^3+^ decay for cycTb-phen. An analysis of the decay curves is presented in Tables S3–S7 using mono- and bi-exponential functions and direct integration of the decay curves. Generally, the decays are not mono-exponential due to the initial faster decay, and the approach to mono-exponential behavior increases with time, as exemplified in the fits to the decay curves after ~0.2 ms. The Eu^3+^ decay in cycEu-phTb, as shown in Fig. 4a, comes closest to the behavior of mono-exponential decay. Taking the values measured after ~0.2 ms following the decay pulse, the lifetimes of the Eu^3+^ ^5^D_0_ excited state in complexes cycTb-phEu and cycEu-phTb decrease slightly from 0.34 to 0.25 ms and from 0.64 to 0.58 ms, respectively, as the temperature increases from 10 to 298 K due to multiphonon relaxation and back-transfer to ^5^D_1_. The Tb^3+^ ^5^D_4_ lifetimes exhibit greater changes: in cycTb-phEu, from 0.42 to 0.11 ms (from 10 K to 298 K) and in cycEu-phTb, from 0.65 to 0.13 ms (from 10 K to 200 K). The changes follow the same patterns as above but are different in magnitude upon considering the values of the “steady-state” lifetime, τ_ST_ (Tables S4 and S7). Additional processes besides multiphonon relaxation are thus involved, such as energy transfer from Tb^3+^ to Eu^3+^.

### Energy transfer from Tb^3+^ to Eu^3+^

There have been many previous literature studies that have investigated this energy transfer with varying concentrations of Tb^3+^ and Eu^3+^ (Table S9). Ideally, this energy transfer can be assessed from a comparison of the luminescence decays of cycTb-phEu and cycTb-phLa; however, since we did not synthesize the latter complex, we considered the decays of cycTb-phEu and cycTb-phen as an alternative estimation. The likely energy transfer pathways^34–40^ are from Tb^3+ 5^D_4_ (20,482 cm^−1^) to Eu^3+ 5^D_1_ (with the lowest crystal field level at 19,048 cm^−1^ as shown in Fig. S5 (literature value at ~19,100 cm^−1^^41^) and to Eu^3+ 5^D_0_ (17,233 cm^−1^, Table S1), with energy differences of 1434 and 3249 cm^−1^, respectively. The participation of ^5^D_1_ in Fig. 3a and b is not evident, particularly when compared with the spectrum of cycLa-phEu (Fig. S5), supporting the direct energy transfer from Tb^3+^ to Eu^3+ 5^D_0_ rather than to ^5^D_1_. From first order selection rules^42^, the pathways of ^5^D_4_–^7^F_6_, ^7^F_5_ involve an electric quadrupole and/or forced electric dipole nonradiative transition. The transition ^7^F_0_–^5^D_0_ is dipole and quadrupole forbidden to first order, although the thermally assisted ^7^F_1_–^5^D_0_ transition is both forced electric dipole and electric quadrupole allowed. Both ^5^D_4_–^7^F_6_ and ^7^F_0_–^5^D_0_ are forbidden by the exchange^43^ selection rule, |*J* - *J*’| = 0, 1 with *J* = 0 ↔ *J*’ = 0 forbidden. The transfer via the charge-transfer state Eu^2+^-Tb^4+^ was not considered.

**Fig. 5:**
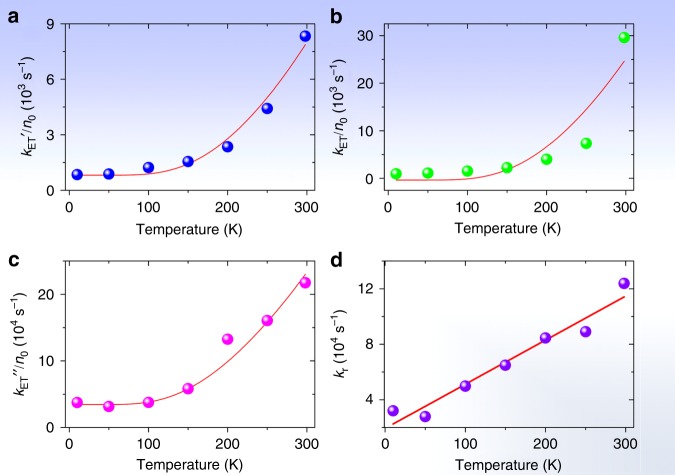
Simulation of experimental values for the Tb^**3+**^ – Eu^**3+**^ energy transfer rate vs. temperature using Eq. 2. (**a**) *k*_ET_’ with values of *a* = 46.9 K^−1^s^−1^ and *b* = 809 s^−1^; **b** *k*_ET_ with values of *a* = 166 K^−1^s^−1^ and *b* = –369 s^−1^. **c** *k*_ET_” with values of *a* = 2793 K^−1^s^−1^ and *b* = 34500 s^−1^. **d** Linear plot of rise time of subtracted decay curves (Table 1) against temperature with a fitting of *y* = (1.93 ± 0.58) + (318 ± 32)*x*, *R*_adj_^2^ = 0.941

The long-term ( > 0.2 ms) luminescence decay constants (i.e., reciprocal lifetimes) of cycTb-phEu (*k*_TbEu_) and cycTb-phen (*k*_Tb_) are listed in Table 1 together with the difference in *k*_TbEu_–*k*_Tb_ = *k*_ET_', taken to indicate the Tb^3+^ → Eu^3+^ energy transfer rate. Alternative descriptions of the energy transfer rate by employing *k*_ET_ and *k*_ET_” are also defined in the table. The magnitude of *k*_Tb_ does not change markedly with temperature (by 10% from 10 K to room temperature). In contrast, *k*_TbEu_ considerably increases by 270% with temperature, and *k*_ET_’ can be described by an exponential growth model (Fig. S8), clearly indicating the importance of temperature in the energy transfer process, which is understood as the thermal population and involvement of ^7^F_1_. Considering only the Eu^3+ 7^F_0_ and ^7^F_1_ *J*-multiplets, with the mean energy of the latter equal to 369 cm^−1^ (from Table S1), and following the description in ref. ^44^, the energy transfer rate can be expressed as:

1 $$k_{{\rm{ET}}}\left( {{\rm{Tb}} \to {\rm{Eu}}} \right) = aN\left( {\,{}^7{\rm{F}}_1} \right){\rm{x}}T + bN\left( {\,{}^7{\rm{F}}_0} \right)$$

2 $$k_{{\rm{ET}}}\left( {{\rm{Tb}} \to {\rm{Eu}}} \right)/N\left( {\,{}^7{\rm{F}}_0} \right) = a \times 3{\rm{exp}}\left( { - 369/kT} \right){\rm{x}}T + b$$

where *a* and *b* are constants; the partition function is given by [1 + 3exp(-369/*kT*)] = *P*, where *k* is the Boltzmann constant; the average ^7^F_1_ energy is 369 cm^−1^; and the populations of ^7^F_1_ and ^7^F_0_ are *N*(^7^F_1_) = *n*_1_ = 3exp(-369/*kT*)/*P* and *N*(^7^F_0_) = *n*_0_ = 1/*P*, respectively. Taking the results from Table 1, Fig. 5a shows a fit of the *k*_ET_’ values from 10 to 298 K with the use of Eq. 2.

**Table 1:** Temperature variation of long-term decay constants, *k*_TbEu_ and *k*_Tb_, for ^5^D_4_ Tb^3+^ emission in cycTb-phEu and cycTb-phen

Temp (K)	Rate constant (s^−1^)					
	*k*_TbEu_	*k*_Tb_	*k*_ET_'	*k*_ET_	*k*_ET_”	*k*_r_
10	2398	1555	843	943	35,507	32,062
50	2421	1545	876	1103	29,165	27,863
100	2740	1523	1217	1493	34,131	49,826
150	3086	1583	1503	2118	51,931	64,851
200	3751	1563	2188	3494	117,128	84,531
250	5618	1687	3931	5858	132,653	89,047
298	8811	1714	7096	21678	159,544	123,854

Temperature variation of Tb–Eu energy transfer rates deduced from the long-term lifetimes (*k*_TbEu_–*k*_Tb_ = *k*_ET_’), direct subtraction of decay curves (1/*τ*_1_ = *k*_ET_, with *τ*_1_ from Table S8), and steady-state lifetimes (1/*τ*_ST_(cycTb-phEu)–1/*τ*_ST_(cycTb-phen) = *k*_ET_”, with *τ*_ST_ from Tables S4 and S7). The steady-state lifetime is determined by integration of the decay curves: $\tau _{{\mathrm{ST}}} = \mathop {\int }\limits_0^\infty \frac{{I(t)}}{{I(0)}}dt,$ refer to the discussion above Table S3. The fitted parameter *k*_r_ = 1/*τ*_2_ is from Table S7

The energy transfer pathway at low temperature, therefore, involves ^7^F_0_ and phonon(s) emission:

$${\!\!\!\!\!\!\!\!\!\!\!}{{}^{5}{\rm{D}}_{4}{\rm{Tb}}^{3 +}\left( {20482} \right) + \, {}^{7}{\rm{F}}_{0}{\rm{Eu}}^{3 + }\left( 0 \right) \to \,{}^{7}{\rm{F}}_{5}{\rm{Tb}}^{3 + }\left( {2019 - 2513} \right)} \\ {+ \, {\rm{Eu}}^{3 + }\, {}^{5}{\rm{D}}_{0}\left( {17233} \right) + {\rm{phonon}}\left( {736 - 1230}\right)}$$

and/or

$${{}^{5}{\rm{D}}_{4}{\rm{Tb}}^{3 + }\left( 20482 \right) + {}^{7}{\rm{F}}_{0}{\rm{Eu}}^{3 + }( 0 ) \to {}^{7}{\rm{F}}_{6}{\rm{Tb}}^{3 + }\left( 0 - 405 \right)} \\ {+ \, {\rm{Eu}}^{3 + }\, {}^{5}{\rm{D}}_{1}\left( 19048 \right) + {\rm{phonon}}\left( 1029 - 1434 \right)}$$

where representative ranges of energies (in cm^−1^) are given in parentheses; or it involves the participation of ^7^F_4_ Tb^3+^ with the absorption of a low energy phonon. The energy transfer rate is higher at room temperature because the forced electric dipole transition, ^7^F_1_ → ^5^D_0_, is involved:

$${}^{5}{\rm{D}}_{4}{\rm{Tb}}^{3 + }\left( {20482} \right) + {}^{7}{\rm{F}}_{1}{\rm{Eu}}^{3 + }\left( {300 - 442} \right) \to {}^{7}{\rm{F}}_{4}{\rm{Tb}}^{3 + }\\ \left( {3289 - 3908} \right) + {\rm{Eu}}^{3 + }\, {}^{5}{\rm{D}}_{0}\left( {17233} \right) \pm {\rm{phonon}}$$

Alternatively, the decay curves of cycTb-phEu and cycTb-phen were directly subtracted (Fig. S9) and the resulting plots were well-fitted by a bi-exponential function (Table S8). The lifetime *τ*_1_ represents the reciprocal of the energy transfer rate from Tb^3+^ → Eu^3+^, *k*_ET_ (listed in Table 1). The values are comparable with those for *k*_ET_’, except that the room temperature value is much higher. Figure 5b shows a plot of the experimental values of *k*_ET_ against inverse temperature and displays a fit using Eq. 2 with a similar result as in Fig. 5a. The fits of the subtracted curves (Fig. S9) produce a rise time, *τ*_2_, in each case, which is equal to the reciprocal of the rate constant, *k*_r_. This rate constant gives a linear plot against temperature, as shown in Fig. 5d, which is expected for the phonon occupation number of a one phonon process. Finally, the plot included in Fig. 5c uses another alternative description of the energy transfer rate, *k*_ET_”, which was derived from steady-state decay lifetimes. Although the numerical values differ, the three fittings serve to confirm the importance of the participation of ^7^F_1_ in the energy transfer process at room temperature.

The energy transfer efficiency from Tb^3+^ → Eu^3+^ (*ƞ*_ET_ = 1– *k*_Tb_/*k*_TbEu_) has very different values when calculated using the three alternative sets of rate constants. The value is above 0.9 and is reasonably independent of temperature when using steady-state lifetimes.

### Energy transfer from antenna to Eu^3+^ ion

The ligand triplet state energy of cycLa-phLa was experimentally determined to be 20,124 cm^−1^ (Fig. 2), which is below the ^5^D_4_ energy (20,482 cm^−1^) of Tb^3+^ in cycTb-phEu. Nevertheless, strong emission is observed from cycTb-phen at room temperature upon excitation into the ligand singlet state to populate the ^5^D_4_ state (Fig. S7). The ^5^D_0_ level (17,233 cm^−1^) of Eu^3+^ lies sufficiently below the triplet state (~3300 cm^−1^) to avoid back-transfer. In addition to triplet T_1_ - metal transfer, there are also opportunities for transfer to the lanthanide ions from higher ligand levels upon 355 nm excitation.

**Fig. 6:**
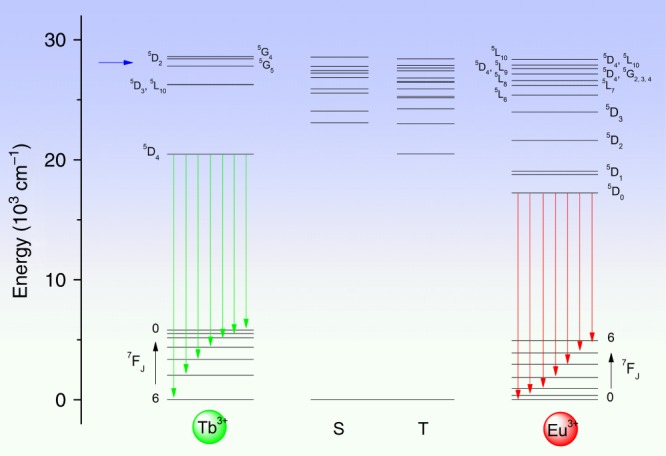
Simplified energy level diagram for Tb^3+^-Eu^3+^ dyads of singlets (S) and triplets (T) of ligand, and lower levels of Tb^3+^ and Eu^3+^ ions. The *J*-multiplet energy levels given for Tb^3+^ and Eu^3+^ may not be exact (and are split by the crystal field) but are illustrative of relative energies. The green and red vertical arrows mark emission from Tb^3+^ and Eu^3+^, respectively. The blue horizontal arrow denotes the 355 nm excitation energy

The calculation of singlet and triplet levels of cycTb-phEu was performed by first optimizing the structure using the Sparkle/RM1 method in MOPAC^28^. Then, the optimized geometry was used in LUMPAC with standard settings to calculate the excited states using Zerner’s intermediate neglect of differential overlap (ZINDO/S) semi-empirical method^30^. Very different triplet state energies resulted when the number of states in the calculation was varied. Figure 2d displays the result with 25 states, where the lowest triplet state energy was calculated at 20,501 cm^−1^ (487.8 nm). In this calculation, there are 25 triplet states and 19 singlet states with transition wavelengths to S_0_ that are longer than 315 nm, with the lowest energy singlets calculated at 391, 416, and 433 nm. The schematic relative energies of the ligand Tb^3+^ and Eu^3+^ levels in cycTb-phEu are shown in Fig. 6 with ligand levels from the above calculation and with the metal ion levels determined experimentally or from other systems. The ability to assign definitive energy transfer pathways is therefore complex.

The 355 nm excitation into the dyad system can populate the ligand level at ~360 nm (Fig. S4), which we associate with the calculated level (Fig. 6) at 28,550 cm^−1^. The gap below this singlet level is spanned by 1 phonon (~770 cm^−1^) so that internal conversion can be fast. In fact, all of the singlet-singlet gaps down to the lowest singlet state calculated at 23,084 cm^−1^ (433 nm) are spanned by one phonon. The latter ligand singlet level is identified with the weak band at 437 nm in Fig. S4. From this figure, it is demonstrated that the rate of internal conversion is slower than the rate of antenna-metal ion energy transfer for these two singlet states. The energy transfer rates from not only these two states, but all of those potentially populated by 355 nm excitation, to the Eu^3+^ levels were investigated using the LUMPAC program, and the results from three different optimizations and calculations are displayed in Tables S11 and S12. The calculated energies represent those from vertical Franck-Condon transitions to unrelaxed excited states. The LUMPAC program neglects intra-ligand intersystem crossing and internal conversion processes. However, it represents the most informative analysis of antenna-metal energy transfer processes currently available. It is evident from Tables S11 and S12 that the major energy transfer pathways from the ligand to Eu^3+^ involve upper Eu^3+^ *J*-multiplets, followed by internal nonradiative relaxation to ^5^D_0_.

### Thermometric properties

**Fig. 7:**
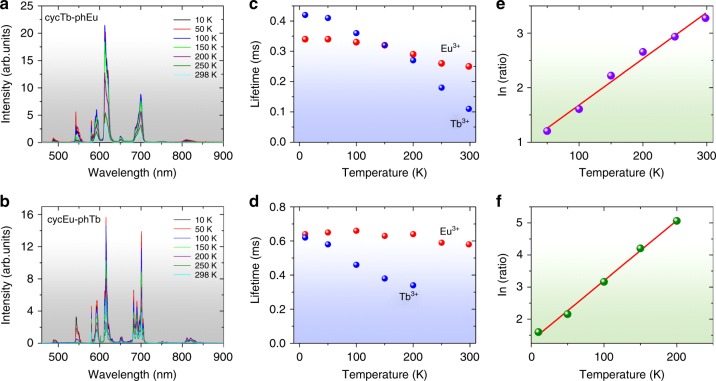
**Emission spectra** (***λ***_**exc**_** = 355 nm) and thermometric properties of complexes.** cycTb-phEu **a** and cycEu-phTb **b** recorded at 10, 50, 100, 150, 200, 250, and 298 K. Temperature-dependent long-term lifetimes of ^5^D_0_ (Eu^3+^) and ^5^D_4_ (Tb^3+^) for cycTb-phEu (**c**) and cycEu-phTb **d**. Natural logarithm of the emission count ratio ^5^D_0_ → ^7^F_4_ (Eu^3+^): ^5^D_4_ → ^7^F_5_ (Tb^3+^) plotted against temperature for **e** cycTb-phEu with fitting equation *y* = (0.83 ± 0.10) + (0.0085 ± 0.0005)*x*, *R*_adj_^2^ = 0.9823 and **f** cycEu-phTb with fitting equation: *y* = (1.32 ± 0.07) ± (0.0188 ± 0.0006)*x*, *R*_adj_^2^ = 0.9960

To investigate their potential application for luminescence thermometry, the temperature-dependent photophysical properties of complexes cycTb-phEu and cycEu-phTb were examined in terms of both the luminescence intensity and lifetime, at intervals between 10 and 298 K (Fig. 7). Three selected temperatures for the emission spectra of these compounds are employed in Figs. S10a, b to show the trends in emission intensity. Moreover, the changes in intensity of the Tb^3+ 5^D_4_ → ^7^F_5_ and Eu^3+ 5^D_0_ → ^7^F_4_ transitions clearly demonstrate the trends. For cycTb-phEu, the major decrease in intensity occurs above ~50 K for Tb^3+^, and it is above ~100 K for Eu^3+^, as shown in Fig. S11a, whereas the decreases begin to occur at lower temperatures for cycEu-phTb (Fig. S11b). The changes in the long-term lifetimes of the ^5^D_4_ and ^7^F_0_ states for these complexes are shown in Fig. 7c, d. These changes are greater for Tb^3+^ than for Eu^3+^.

The different temperature-dependent luminescent emission of the ^5^D_4_ → ^7^F_5_ transition of Tb^3+^ and the ^5^D_0_ → ^7^F_4_ transition of Eu^3+^ indicates that these dinuclear complexes are potential self-referencing thermosensitive probes that do not require any cumbersome calibration of the emission intensity. This makes a luminescent thermometer more reliable. The two target emission intensity measurements lie in clear spectral windows so that background interference is absent. The ratio of the emission intensities, *R*, follows an exponential growth curve with temperature (Fig. S12), and the natural logarithm of the ratio is plotted against temperature in Fig. 7e, f with cycEu-phTb providing the better fit. The relative sensitivity is *S* = (d*R*/d*T*)/*R*, i.e., the change in ratio with temperature divided by the value of the ratio. Since, from Fig. S12b:

3 $${{\hskip -8pt}R = A\,{\rm{x}}\,{\rm{exp}}\left( {B{\rm{x}}{\it{T}}} \right){\rm{,where}}\,{{A = 3}}{\rm{.96}}\,{\rm{and}}\,{{B = 0}}{\rm{.01857K}}^{{ - 1}}}$$

4 $${\hskip -8pt}S = {\mathrm{B}}$$

and the relative sensitivity is constant at 1.86%K^−1^. The temperature resolution, d*T*, is given by:

$${\mathrm{d}}T = \left( {1/S} \right)\times \left( {{{d}}R/R} \right)$$

where d*R* is the error (standard deviation) in *R* value at temperature *T* and is < 1 °C at *T* > 200 K (Fig. S12c).

The dyad cycEu-phTb is therefore suitable for use as a luminescent self-referencing thermo-monitor with temperature responsive luminescence and lifetimes.

## Discussion

A strategy to synthesize hetero-dinuclear complexes that consist of chemically similar lanthanides has been introduced by which cycTb-phEu and cycEu-phTb were produced. The different energy gaps between the ligand triplet state and the acceptor lanthanide ion states, as well as the Tb^3+^ to Eu^3+^ energy transfer, result in different luminescence performances for each metal ion, giving an increased temperature-dependent luminescent emission ratio for europium over terbium. Both dinuclear complexes illustrated excellent temperature sensitivity over a wide temperature range, with cycEu-phTb having the best potential as an optical thermometer using an excitation wavelength of 355 nm. The temperature-dependent energy transfer between the two lanthanide ions that quenches Tb^3+^ emission has been rationalized by observing the importance of the ^7^F_1_ state at room temperature. LUMPAC calculations point to the energy transfer to Eu^3+^ from higher ligand states, rather than from the lowest triplet state.

## Materials and methods

All chemicals were purchased and received without further purification. NMR spectra were measured on a Bruker400 (400 Hz) magnetic resonance spectrometer with chemical shifts expressed as parts per million (ppm) and coupling constants, *J*, as Hertz (Hz). Mass spectrometry was taken on an ABI QSTAR Elite quadrupole-time-of-flight mass spectrometer using electrospray ionization (ESI) as the ion source. The HPLC measurements were conducted on an Agilent 1200 series HPLC (Column: Vision HT C18 HL 5u, length 250 mm, Serial No. 5151920 ID 4.6 mm). Fourier transform-infrared spectroscopy was performed on a PerkinElmer Paragon 1000 PC FT-IR spectrometer with KBr tablets. The emission spectra and decay lifetime measurements were recorded on a Horiba fluorescence spectrometer with a xenon lamp and a SpectreLED as the excitation sources, on an iHR550 spectrometer with a Nd^3+^:YAG laser as the excitation source, and on a Mini-tau from Edinburgh Instruments.

## Electronic supplementary material

Supplemental Material

## References

[1] Miyata K (2013). Chameleon luminophore for sensing temperatures: control of metal-to-metal and energy back transfer in lanthanide coordination polymers. Angew. Chem. Int Ed..

[2] Hatanaka M (2017). Organic linkers control the thermosensitivity of the emission intensities from Tb(III) and Eu(III) in a chameleon polymer. Chem. Sci..

[3] Nakano M (2017). Genetically encoded ratiometric fluorescent thermometer with wide range and rapid response. PLoS ONE.

[4] Wang XD, Wolfbeis OS, Meier RJ (2013). Luminescent probes and sensors for temperature. Chem. Soc. Rev..

[5] Cui YJ (2012). A luminescent mixed-lanthanide metal-organic framework thermometer. J. Am. Chem. Soc..

[6] Marciniak L, Prorok K, Francés-Soriano L, Pérez-Prieto J, Bednarkiewicz A (2016). A broadening temperature sensitivity range with a core-shell YbEr@YbNd double ratiometric optical nanothermometer. Nanoscale.

[7] N’Dala-Louika I (2017). Ratiometric mixed Eu-Tb metal-organic framework as a new cryogenic luminescent thermometer. J. Mater. Chem. C..

[8] Lu HY (2016). Stark sublevels of Er^3+^-Yb^3+^ codoped Gd_2_(WO_4_)_3_ phosphor for enhancing the sensitivity of a luminescent thermometer. RSC Adv..

[9] Wang HZ, Zhao D, Cui YJ, Yang Y, Qian GD (2017). A Eu/Tb-mixed MOF for luminescent high-temperature sensing. J. Solid State Chem..

[10] Zhou J, Xia ZG, Bettinelli M, Liu QL (2016). Photoluminescence tuning via energy transfer in Eu-doped Ba_2_(Gd,Tb)_2_Si_4_O_13_ solid-solution phosphors. RSC Adv..

[11] Morrison G, Latshaw AM, Spagnuolo NR, Zur Loye HC (2017). Observation of intense X-ray scintillation in a family of mixed anion silicates, Cs_3_RESi_4_O_10_F_2_ (RE = Y, Eu-Lu), obtained via an enhanced flux crystal growth technique. J. Am. Chem. Soc..

[12] Romanova KA, Freidzon AY, Bagaturyants AA, Galyametdinov YG (2014). Ab initio study of energy transfer pathways in dinuclear lanthanide complex of europium(III) and terbium(III) ions. J. Phys. Chem. A.

[13] Bao GC (2018). Reversible and sensitive Hg^2+^ detection by a cell-permeable ytterbium complex. Inorg. Chem..

[14] Yu YL (2018). Self-calibrating optic thermometer based on dual-emission nanocomposite. J. Alloy. Compd..

[15] Dai ZC (2014). Ratiometric time-gated luminescence probe for hydrogen sulfide based on lanthanide complexes. Anal. Chem..

[16] Yang J, Zhang CM, Li CX, Yu YN, Lin J (2018). Energy transfer and tunable luminescence properties of Eu^3+^ in TbBO_3_ microspheres via a facile hydrothermal process. Inorg. Chem..

[17] Rao XT (2013). A highly sensitive mixed lanthanide metal-organic framework self-calibrated luminescent thermometer. J. Am. Chem. Soc..

[18] Tanaka F, Ishibashi T (1996). Energy transfer between lanthanide ions in dinuclear complexes. J. Chem. Soc., Faraday Trans..

[19] Zhong Q (2016). Novel stoichiometrically erbium−ytterbium cocrystalline complex exhibiting enhanced near-infrared luminescence. Inorg. Chem..

[20] Liu X, Zhu J, Ni HT, Ma B, Liu L (2016). Luminescent properties of a polymer photoluminescent composite containing the binuclear (Eu, Tb) complex as an emitter. J. Macromol. Sci., Part B.

[21] Nonat A, Liu T, Jeannin O, Camerel F, Charbonnierè LJ (2018). Energy transfer in supramolecular heteronuclear lanthanide dimers and application to fluoride sensing in water. Chem. Eur. J..

[22] Debroye E (2014). Controlled synthesis of a novel heteropolymetallic complex with selectively incorporated lanthanide(III) ions. Inorg. Chem..

[23] Natrajan, L. S., Villaraza, A. J. L., Kenwright, A. M., Faulkner S. Controlled preparation of a heterometallic lanthanide complex containing different lanthanides in symmetrical binding pockets. *Chem. Comm.* 6020–6022 (2009). 10.1039/b913702e 19809630

[24] Grimme S, Brandenburg JG, Bannwarth C, Hansen A (2015). Consistent structures and interactions by density functional theory with small atomic orbital basis sets. J. Chem. Phys..

[25] Weigend F, Ahlrichs R (2005). Balanced basis sets of split valence, triple zeta valence and quadruple zeta valence quality for H to Rn: design and assessment of accuracy. Phys. Chem. Chem. Phys..

[26] Dolg M, Stoll H, Preuss H (1989). Energy‐adjusted *ab initio* pseudopotentials for the rare earth elements. J. Chem. Phys..

[27] Neese F (2012). The ORCA program system. Wiley Interdiscip. Rev: Comput. Mol. Sci..

[28] Stewart JJP (2004). Use of semiempirical methods for detecting anomalies in reported enthalpies of formation of organic compounds. J. Phys. Chem. Ref. Data.

[29] Rocha GB, Freire RO, Simas AM, Stewart JJP (2006). RM1: a reparameterization of AM1 for H, C, N, O, P, S, F, Cl, Br, and I. J. Comput. Chem..

[30] Dutra JDL, Bispo TD, Freire RO (2014). LUMPAC lanthanide luminescence software: efficient and user friendly. J. Comput. Chem..

[31] Malta OL (2008). Mechanisms of non-radiative energy transfer involving lanthanide ions revisited. J. Non-Cryst. Solids.

[32] Pitchaimani P, Lo KM, Elango KP (2015). Synthesis, spectral characterization, crystal structures of lanthanide(III) pyrrolidine dithiocarbamate complexes and their catalytic activity. J. Coord. Chem..

[33] Eliseeva SV, Bünzli JCG (2010). Lanthanide luminescence for functional materials and bio-sciences. Chem. Soc. Rev..

[34] Sato S (2015). Luminescence of fusion materials of polymeric chain-structured lanthanide complexes. Polym. J..

[35] Fomina IG (2013). Synthesis and characterization of new heterodinuclear (Eu, Tb) lanthanide pivalates. Polyhedron.

[36] Irfanullah M, Iftikhar K (2013). Synthesis and spectroscopic analysis of an extended series of hetero dinuclear complexes containing two different lanthanides in 1:1 stoichiometry. Inorg. Chim. Acta.

[37] Anh TK (1988). Energy transfer between Tb^3+^ and Eu^3+^ in Y_2_O_3_ crystals. J. Lumin.

[38] Carrasco I, Piccinelli F, Bettinelli M (2017). Luminescence of Tb-based materials doped with Eu^3+^: case studies for energy transfer processes. J. Lumin..

[39] Hou ZY (2011). Electrospinning-derived Tb_2_(WO_4_)_3_: Eu^3+^ nanowires: energy transfer and tunable luminescence properties. Nanoscale.

[40] Moran DM, May PS, Richardson FS (1994). Measurement and analysis of electronic energy transfer between Tb^3+^ and Eu^3+^ ions in Cs_2_NaY_1−x−y_ Tb_x_Eu_y_Cl_6_. Chem. Phys..

[41] Binnemans K (2015). Interpretation of europium(III) spectra. Coord. Chem. Rev..

[42] Tanner PA, Duan CK (2010). Luminescent lanthanide complexes: selection rules and design. Coord. Chem. Rev..

[43] Dexter DL (1953). A theory of sensitized luminescence in solids. J. Chem. Phys..

[44] Laulicht I, Meirman S, Ehrenberg B (1984). Fluorescent linewidths and excitation transfer in Eu_0.33_Tb_0.66_P_5_O_14_ crystals. J. Lumin..

