# Supplementary material for: A stoichiometric terbium-europium dyad molecular thermometer: energy transfer properties
Source: Light Sci Appl. 2018 Nov 28;7:96. doi: 10.1038/s41377-018-0097-7 (PMC6258678; doi:10.1038/s41377-018-0097-7)
Supplement: Supplementary file 1 — Supplemental Material [file 41377_2018_97_MOESM1_ESM.docx]

**SUPPLEMENTARY INFORMATION**

A Stoichiometric Terbium-Europium Dyad Molecular Thermometer: Energy Transfer Properties

Guochen Bao^1,2,3^, Ka-Leung Wong^1^, Dayong Jin^2,3^ and Peter A. Tanner^1^

^1^Department of Chemistry, Hong Kong Baptist University, Kowloon Tong, Hong Kong SAR, People’s Republic of China

^2^Institute for Biomedical Materials and Devices (IBMD), Faculty of Science, University of Technology Sydney, Sydney, New South Wales 2007, Australia

^3^School of Mathematical and Physical Science, Faculty of Science, University of Technology Sydney, Sydney, New South Wales 2007, Australia

contents

[**Structures and synthetic scheme** 2](#_Toc522797893)

[**Synthetic procedures** 3](#_Toc522797894)

[**Photophysical properties** 5](#_Toc522797895)

[**Calculations** 18](#_Toc522797896)

[**NMR, MS and HPLC results** 19](#_Toc522797897)

[**References** 27](#_Toc522797898)

**Structures and synthetic scheme**

**
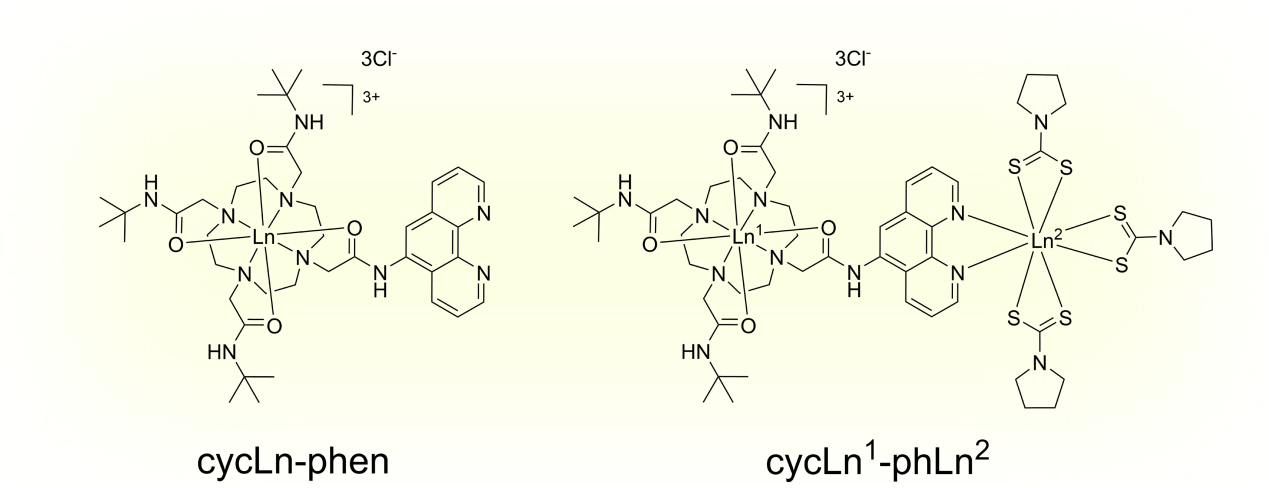
**

Fig. S1. Structures and naming of the compounds employed in this work**: cycLn-phen, cycLn^1^-phLn^2^,** where Ln represents a lanthanide element.


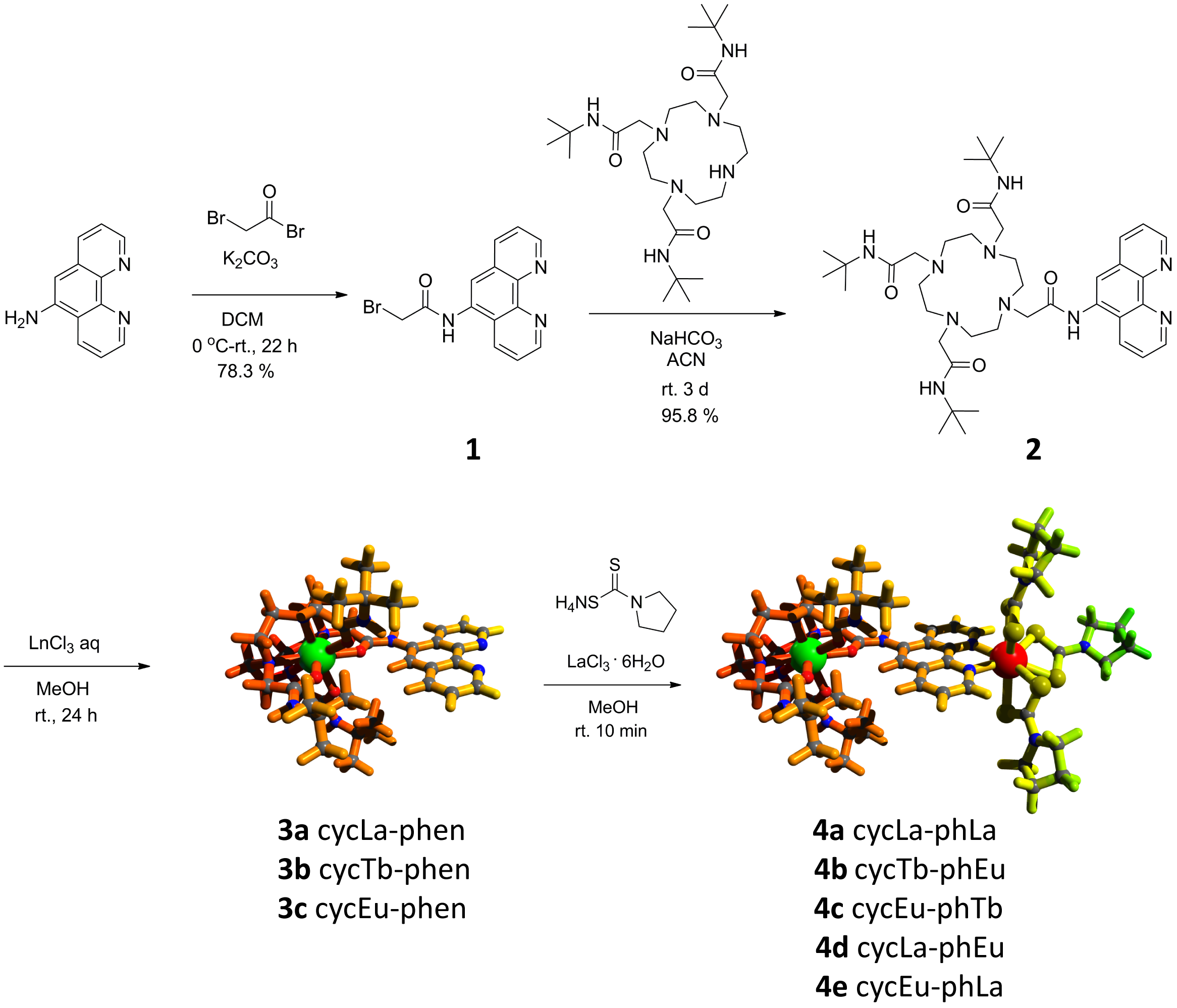


Scheme S1. Synthesis of ligands and complexes.

**Synthetic procedures**

**Synthesis of 2-bromo-N-(1,10-phenanthrolin-5-yl)acetamide 1.** K_2_CO_3_ ( 5.53 g, 40.96 mmol) was added to a suspension of 1,10-phenanthrolin-5-amine (2.0 g, 10.24 mmol) in 500 mL of DCM. 2-Bromoacetyl bromide (1.3 mL, 15.36 mmol) was added dropwise at 0 °C and the mixture was stirred at room temperature for 22 h. The reaction content was then quenched with 20 mL of water and stirred for another 10 min. The yellow precipitate was collected by filtration, washed with small amount of DCM, saturated NaHCO_3_ aqueous and water, then dried under vacuum to afford product as yellow solid (2.48 g, 78.3% yield). ^1^H NMR (400 MHz, DMSO-d_6_) δ 10.60 (s, 1H), 9.15 (dd, *J* = 4.2, 1.6 Hz, 1H), 9.06 (dd, *J* = 4.3, 1.7 Hz, 1H), 8.61 (dd, *J* = 8.4, 1.5 Hz, 1H), 8.50 (dd, *J* = 8.1, 1.6 Hz, 1H), 8.18 (s, 1H), 7.86 (dd, *J* = 8.4, 4.3 Hz, 1H), 7.76 (dd, *J* = 8.1, 4.3 Hz, 1H), 4.29 (s, 2H). ^13^C NMR (101 MHz, DMSO-d_6_) δ = 166.26, 150.05, 149.63, 136.18, 136.10, 131.4, 131.06, 127.97, 124.53, 123.72, 123.02, 120.55, 30.02. ESI-MS: calcd. for [M + H^+^]^+^ = 316.0080, found: 316.0078; calcd. for [M + Na^+^]^+^ = 337.9899, found: 337.9902.

**Synthesis of 2,2',2''-(10-(2-((1,10-phenanthrolin-5-yl)amino)-2-oxoethyl)-1,4,7,10- tetraazacyclododecane-1,4,7-triyl)tris(N-(tert-butyl)acetamide) (cyc-phen) 2**. 2,2',2''-(1,4,7,10-tetraazacyclododecane-1,4,7-triyl)tris(N-(tert-butyl)acetamide) was added to a solution of 2-bromo-N-(1,10-phenanthrolin-5-yl)acetamide **1** (1 g, 3.16 mmol) and NaHCO_3_ (3.24 mmol) in 200 mL of MeCN at room temperature. The reaction content was stirred at room temperature for 3 days. Then the solid was filtered off and washed with small amount of methanol. The organic phases were combined and the solvent of the organic phase was removed to give the crude product. The crude product was washed with MeCN in water bath (60 ^o^C ) in ultrasound condition for 10 min. The yellow precipitate was collected by filtration and washed with 10 mL of MeCN and dried under vacuum to give yellow product as solid (2.260 g, 95.8% yield). ^1^H NMR (400 MHz, DMSO-d_6_) δ 10.35 (s, 1H), 9.10 (dd, *J* = 4.2, 1.5 Hz, 1H), 9.02 (dd, *J* = 4.2, 1.6 Hz, 1H), 8.64 (dd, *J* = 8.4, 1.5 Hz, 1H), 8.23 (d, *J* = 6.8 Hz, 1H), 8.04 (s, 1H), 7.78 – 7.71 (m, 2H), 7.67 (s, 3H), 2.90 (br, 13H), 2.27 (br, 11H), 1.27 (s, 9H), 1.11 (s, 18H). ^13^C NMR (101 MHz, DMSO-d_6_) δ = 171.43, 170.48, 170.18, 149.80, 149.32, 149.29, 145.76, 143.87, 135.29, 132.29, 127.90, 124.83, 123.54, 122.65, 120.25, 57.61, 57.11, 56.22, 50.33, 50.22, 28.28, 28.19. ESI-MS: calcd: [M + H^+^] = 747.5028, found: 747.5046. [M + Na^+^]^+^ = 769.4848, found: 769.4743. [M + Na^+^ + H^+^]^2+^/2 = 385.2460, found: 385.2444.

**Synthesis of compound cycLa-phen 3a.** The solution of LaCl_3_•6H_2_O (142 mg, 0.4 mmol ) in 40 mL of deionized H_2_O was added to a solution of **2** (299 mg, 0.4 mmol) in 40 mL of MeOH. The reaction mixture was stirred at room temperature for 24 h. Then removed half of the solvent and the remained content was washed with DCM (2 × 20 mL). The solvent was removed and the crude product was recrystallized with MeOH and Et_2_O to give yellow product as solid (400 mg, 99.0% yields). ^1^H NMR (400 MHz, DMSO-d_6_) δ 11.82 (s, 1H), 9.11 (m, 5H), 8.71 (d, *J* = 8.2 Hz, 1H), 8.38 (d, *J* = 7.6 Hz, 1H), 8.02 (s, 1H), 7.84 (m, 2H), 3.39 - 4.12 (br, 12H), 1.96 - 3.07 (br, 12H), 1.24 (s, 9H), 1.14 (s, 18H). ESI-MS: calcd. for [M - 3Cl^-^ - 2H^+^]^+^ = 883.3857, found: 883.3858.

**Synthesis of compound cycTb-phen 3b**. The solution of TbCl_3_•6H_2_O (112 mg, 0.3 mmol ) in 30 mL of deionized H_2_O was added to a solution of **2** (224 mg, 0.3 mmol) in 30 mL of MeOH. The reaction content was stirred at room temperature for 24 h. Then removed half of the solvent and the remained content was washed with DCM (2 × 20 mL). The solvent was the removed and the crude product was recrystallized with MeOH and Et_2_O to give yellow product as solid (299 mg, 96.8% yield). ESI MS: calcd. for [M - 3Cl^-^ - 2H^+^]^+^ = 903.8938, found: 903.4052.

**Synthesis of compound cycEu-phen 3c**. A solution of EuCl_3_•6H_2_O (146 mg, 0.4 mmol ) in 40 mL of deionized H_2_O was added to a solution of **2** (299 mg, 0.4 mmol) in 40 mL of MeOH. The reaction was stirred at room temperature for 24 h. Then removed half of the solvent and the remained content was washed with DCM (2 ×20 mL). The solvent was the removed and the crude product was recrystallized with MeOH and Et2O to give yellow product as solid (400 mg, 97.8% yield). mp. > 300 ^o^C. ESI MS: calcd. for [M - 3Cl^-^ - 2H^+^]^+^ = 897.4000, found: 897.4065.

**Synthesis of compound cycLa-phLa 4a**. A mixture of **3a** (101 mg, 0.10 mmol) and ammonium pyrrolidine-1-carbodithioate (49 mg, 0.30 mmol) in 10 mL of MeOH were added to a solution of LaCl_3_•6H_2_O (35 mg, 0.10 mmol) in 5 mL of MeOH dropwise. Then the reaction mixture was stirred for 10 min at room temperature. Half of the solvent was removed and the left solution was transferred to a 50 mL of centrifugal tube. 30 mL of Et_2_O was added dropwise and yellow solid was precipitated. The suspension was centrifuged and the organic phase was removed. The precipitation and centrifugation were repeated twice. The precipitate was dried under vacuum to give product as yellow solid (141 mg, 88.7% yield). ^1^H NMR (400 MHz, DMSO-d_6_) δ 9.09 (m, 5H), 8.71 (d, *J* = 8.1 Hz, 1H), 8.34 (d, *J* = 7.5 Hz, 1H), 7.94 (s, 1H), 7.81 (m, 3H), 3.90 (br, 3H), 3.64 (t, *J* = 6.8 Hz, 12H), 3.45 (br, 8H), 2.61 (br, 8H), 2.20 (br, 5H), 1.96 – 1.71 (m, 12H), 1.24 (s, 9H), 1.15 (s, 18H). HPLC: retention time: 15.52 min. calcd. for [M - La^3+^ - 3Cl^-^ - H^+^ - 3pdtc^-^]^2+^/2 = 442.1965, found: 442.1888.

**Synthesis of compound cycTb-phEu 4b.** A mixture of **3b** (155 mg, 0.15 mmol) and ammonium pyrrolidine-1-carbodithioate (74 mg, 0.45 mmol) in 10 mL of MeOH were added to a solution of EuCl_3_•6H_2_O (55 mg, 0.15 mmol) in 5 mL of MeOH dropwise. Then the reaction was stirred for 10 min at room temperature. Half of the solvent was removed and the left solution was transferred to a 50 mL of centrifugal tube. 50 mL of Et_2_O was added dropwise and yellow solid was precipitated. The suspension was centrifuged and the organic phase was removed. The precipitation and centrifugation were repeated twice. The precipitate was dried under vacuum to give product as yellow solid (223 mg, 91.8% yield). calcd. for [M - 3Cl^-^ - H^+^ -Eu^3+^ - 3pdtc^-^]^2+^/2 = 452.4508, found: 452.2040.

**Synthesis of compound cycEu-phTb 4c.** A mixture of **3c** (153 mg, 0.15 mmol) and ammonium pyrrolidine-1-carbodithioate (74 mg, 0.45 mmol) in 10 mL of MeOH were added to a solution of TbCl_3_•6H_2_O (56 mg, 0.15 mmol) in 5 mL of MeOH dropwise. The reaction content was stirred for 10 min at room temperature. Half of the solvent was removed and the left solution was transferred to a 50 mL of centrifugal tube. 50 mL of Et_2_O was added dropwise and yellow solid was precipitated. The suspension was centrifuged and the organic phase was removed. The precipitation and centrifugation were repeated twice. The precipitate was dried under vacuum to give product as yellow solid (230 mg, 94.6% yield). ESI MS: calcd for [M -3Cl^-^- Tb^3+^ - 3pdtc^-^ - 2H^+^]^+^ = 897.4065, found: 897.3974.

**Synthesis of compound cycLa-phEu 4d.** A mixture of **3a** (151.5 mg, 0.15 mmol) and ammonium pyrrolidine-1-carbodithioate (74 mg, 0.45 mmol) in 10 mL of MeOH was added dropwise to a solution of EuCl_3_**^.^**6H_2_O (55 mg, 0.15 mmol) in 5 mL of MeOH. Then the reaction was stirred for 10 min at room temperature. Half of the solvent was removed and the left solution was transferred to a 50 mL of centrifugal tube. 40 mL of Et_2_O was added dropwise and yellow solid was precipitated. The suspension was centrifuged and the organic phase was removed. Then repeated the precipitation and centrifugation twice. The participate was dried under vacuum to give product as yellow solid (229 mg, 95.4% yield). mp. > 300 ^o^C. ^1^H NMR (400 MHz, DMSO-d_6_) δ 9.10 (d, *J* = 32.0 Hz, 5H), 8.70 (s, 1H), 8.33 (s, 1H), 7.90 (s, 1H), 7.80 (s, 2H), 3.69 (s, 12H), 2.51 - 3.32 (br, 18H), 2.02 - 2.42 (br, 6H), 1.89 (s, 12H), 1.24 (s, 9H), 1.16 (s, 18H).

**Synthesis of compound cycEu-phLa 4e**. A mixture of cyclenEu-ph **3c** (153.5 mg, 0.15 mmol) and ammonium pyrrolidine-1-carbodithioate (74 mg, 0.45 mmol) in 10 mL of MeOH was added dropwise to a solution of LaCl_3_**^.^**6H_2_O (55 mg, 0.15 mmol) in 5 mL of MeOH. Then the reaction was stirred for 10 min at room temperature. Half of the solvent was removed and the left solution was transferred to a 50 mL of centrifugal tube. 40 mL of Et_2_O was added dropwise and yellow solid was precipitated. The suspension was centrifuged and the organic phase was removed. The precipitation and centrifugation were repeated twice. The precipitate was dried under vacuum to give product as yellow solid (240 mg, 99.9% yield). ESI MS: calcd for [M -3Cl^-^- La^3+^ - 3pdtc^-^ - 2H^+^]^+^ = 897.4006, found: 897.3903.

**Photophysical properties**


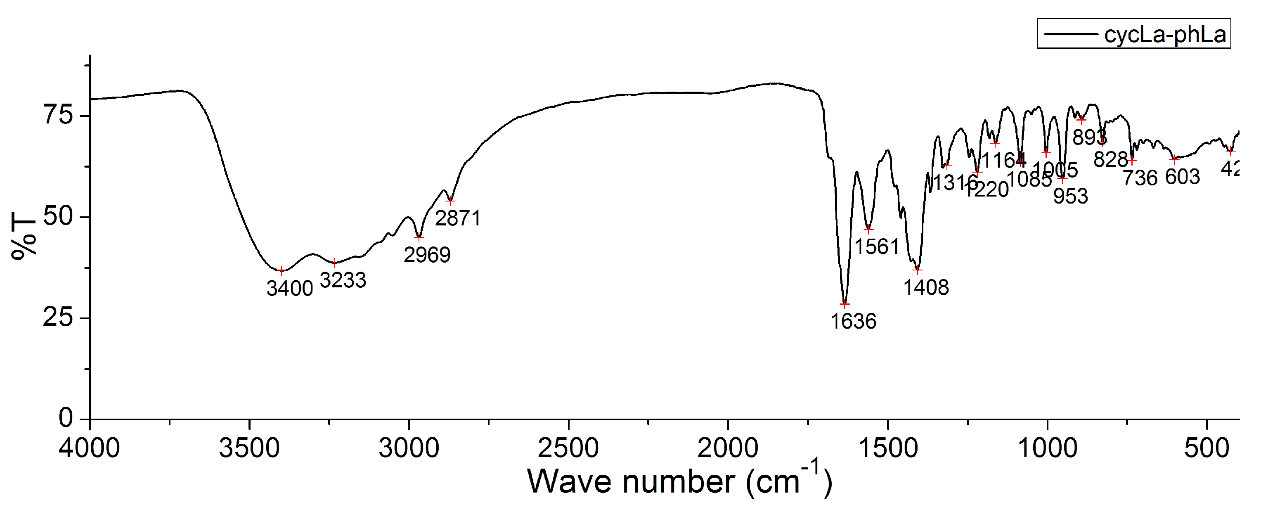


Fig. S2. FT-IR spectrum of complex **cycLa-phLa** **4a**.


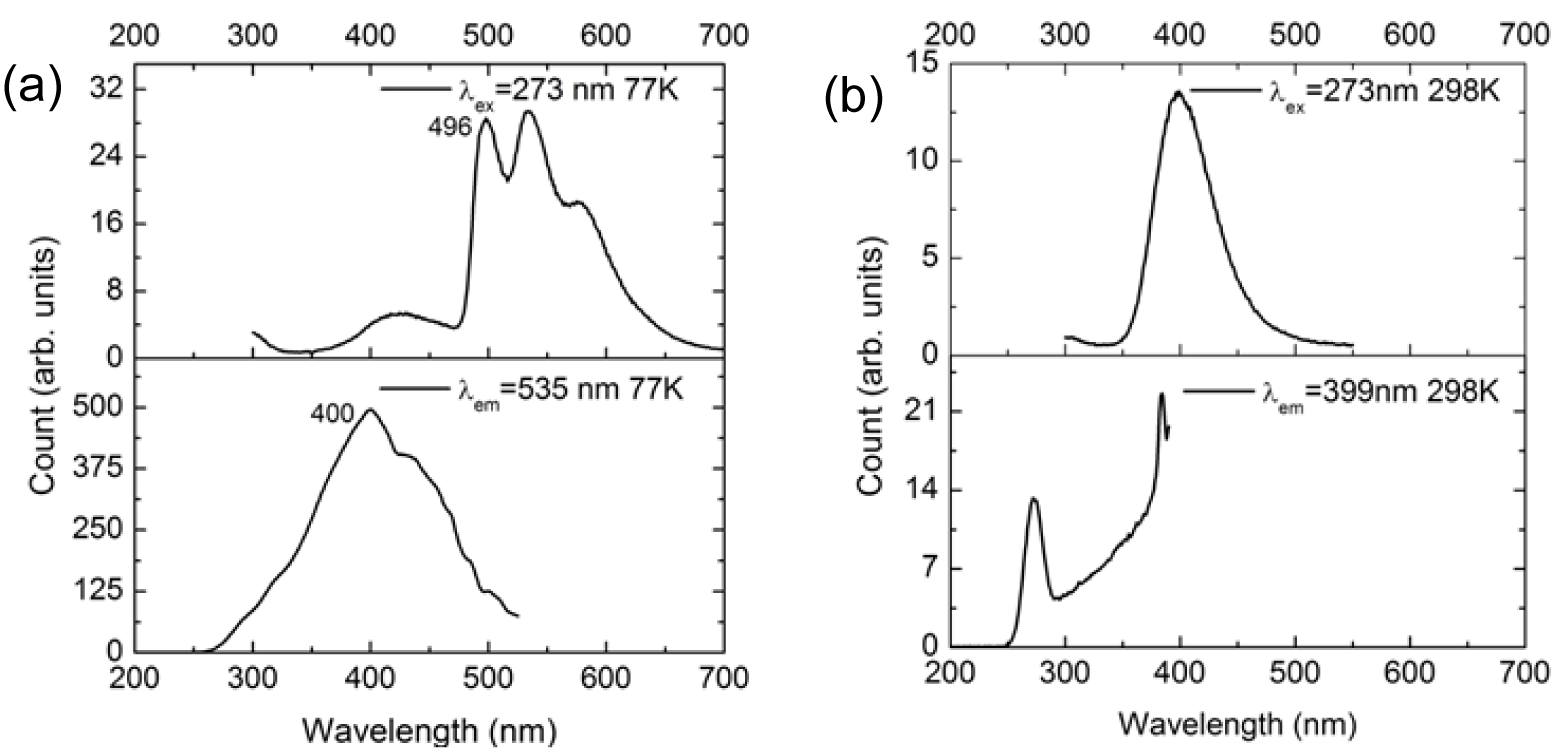


Fig. S3. (a) Triplet emission and excitation spectra of **cycLa-phLa** in the solid state at 77 K. (b) Singlet emission and excitation spectrum of **cycLa-phLa** at 298 K in the solid state.


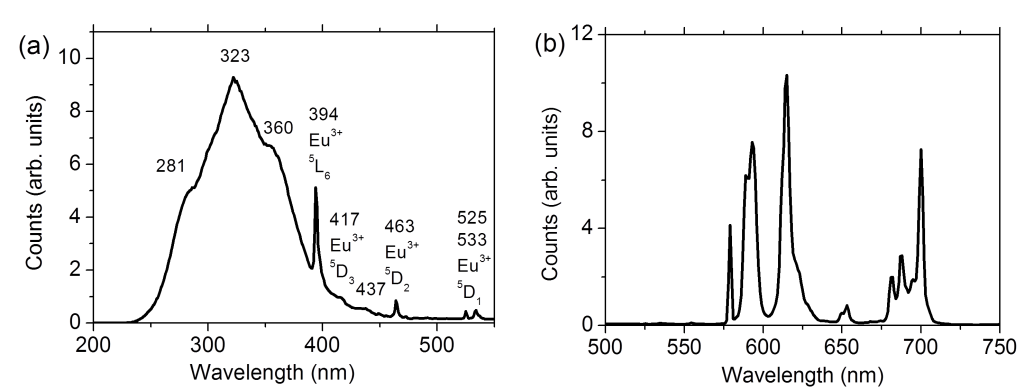


Fig. S4. Excitation (λ_em_ 615 nm) and emission (λ_exc_ 321 nm) of **cycEu-phLa** in the solid phase at room temperature. Note the weak ^5^D_1_ emission in this system.


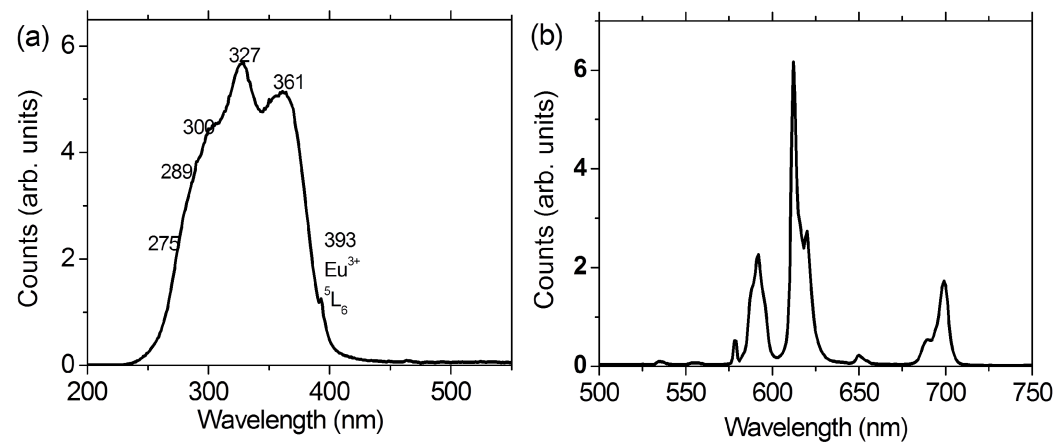


Fig. S5. Excitation and emission of **cycLa-phEu** in the solid phase at room temperature. Note the weak ^5^D_1_ emission in this system. ^5^D_1_ → ^7^F_0_ is at 525 nm (19048 cm^-1^) and bands are observed at 534.6, 552.8 and 558.4 nm (i.e., 342, 948 and 1140 cm^-1^ to lower energy).


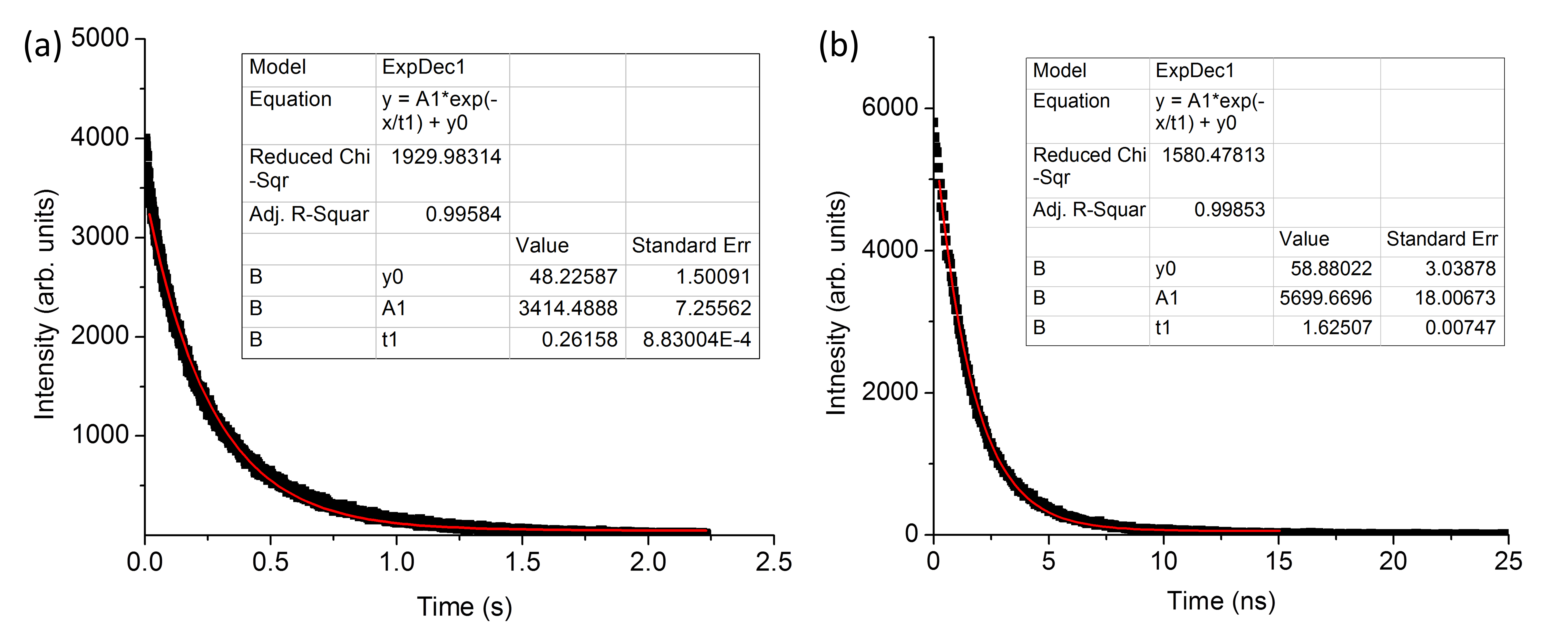


Fig. S6. (a) Decay of triplet emission of **cycLa-phLa** at 77 K (λ_exc_ 355 nm; λ_em_ 550 nm). τ = 0.26 s. (b) Decay of singlet emission of **cycLa-phLa** at 400 nm at room temperature. (λ_exc_ 365 nm; λ_em_ 400 nm). τ = 1.6 ns.





Figure S7. 298 K Emission and excitation spectra of **cycTb-phen**.

The contribution to absorption by the 4f^8^ – 4f^8^ absorption bands of Tb^3+^ is negligible compared with ligand absorption. This is well-demonstrated by the excitation spectrum of **cycTb-ph** (Fig. S7) where no fine structure is visible.

Table S1. 10 K emission spectrum of **cycTb-phEu (Fig. 3a) and cycTb-phen (Fig. 3c).**

| **cycTb-phEu** | | | | | | **cycTb-phen** | | | |
| --- | --- | --- | --- | --- | --- | --- | --- | --- | --- |
| Wavelength (nm) | Energy  (cm^-1^) | Tb^3+^  Transition | Tb^3+^  CF level (cm^-1^) | Eu^3+^  Transition | Eu^3+^  CF level (cm^-1^) | Wavelength (nm) | Energy  (cm^-1^) | Tb^3+^  Transition | Tb^3+^  CF level (cm^-1^) |
|  |  |  |  |  |  | 488.23 | 20482 | ^5^D_4_→^7^F_6_ | 20482/0 |
| 485.63 sh | 20592 | ^5^D_4_→^7^F_6_ | 20592 |  |  | 490.87 | 20372 |  | 110 |
| 488.24 | 20482 |  | 20482/0 |  |  | 491.77 | 20335 |  | 147 |
| 491.03 | 20365 |  | 117 |  |  | 493.73 | 20254 |  | 228 |
| 492.48 | 20305 |  | 177 |  |  | 495.85 | 20167 |  | 315 |
| 498.09 | 20077 |  | 405 |  |  | 498.26 | 20070 |  | 412 |
| 541.61 sh | 18463 | ^5^D_4_→^7^F_5_ | 2019 |  |  | 541.68 sh | 18461 | ^5^D_4_→^7^F_5_ | 2021 |
| 542.68 | 18427 |  | 2055 |  |  | 542.47 | 18434 |  | 2048 |
| 545.35 | 18337 |  | 2145 |  |  | 545.18 | 18343 |  | 2139 |
| 547.03 | 18281 |  | 2201 |  |  | 546.65 | 18293 |  | 2189 |
| 548.41 | 18235 |  | 2247 |  |  | 548.3 sh | 18238 |  | 2244 |
| 550.88 b | 18153 |  | 2329 |  |  | 551.47 b | 18133 |  | 2329 |
| 556.51 | 17969 |  | 2513 |  |  | 556.59 | 17967 |  | 2515 |
| 580.28 | 17233 |  |  | ^5^D_0_→^7^F_0_ | 17233/0 | 581.64 sh | 17193 | ^5^D_4_→^7^F_4_ | 3289 |
| 584.33 | 17114 | ^5^D_4_→^7^F_4_ | 3368 |  |  | 583.78 | 17130 |  | 3352 |
| 590.58 | 16933 |  |  | ^5^D_0_→^7^F_1_ | 300 | 587.97 | 17008 |  | 3474 |
| 592.79 | 16869 |  |  |  | 364 | 592.62 | 16874 |  | 3608 |
| 595.54 | 16791 |  |  |  | 442 | 603.36 | 16574 |  | 3908 |
| 612.80 | 16319 |  |  | ^5^D_0_→^7^F_2_ | 914 | 620.78 | 16109 | ^5^D_4_→^7^F_3_ | 4373 |
| 614.33 | 16278 |  |  |  | 955 | 624.00 | 16026 |  | 4456 |
| 617.41 | 16197 |  |  |  | 1036 | 627.63 | 15933 |  | 4549 |
| 620.49 | 16116 |  |  |  | 1117 | 631.25 | 15842 |  | 4640 |
| 650.84 | 15365 |  |  | ^5^D_0_→^7^F_3_ | 1868 | 644.98 | 15504 |  | 4978 |
| 687.08 | 14554 |  |  | ^5^D_0_→^7^F_4_ | 2679 | 652.30 | 15330 | ^5^D_4_→^7^F_2_ | 5152 |
| 694.30 | 14403 |  |  |  | 2830 | 656.12 | 15241 |  | 5241 |
| 700.36 | 14278 |  |  |  | 2955 | 662.35 | 15098 |  | 5384 |
| 743.45 | 13451 |  |  | ^5^D_0_→^7^F_5_ | 3782 | 667.72 | 14976 | ^5^D_4_→^7^F_1_ | 5506 |
| 747.25 | 13382 |  |  |  | 3851 | 674.32 | 14830 |  | 5652 |
| 751.24 | 13311 |  |  |  | 3922 | 680.92 | 14686 |  | 5796 |
| 806.54 | 12399 |  |  | ^5^D_0_→^7^F_6_ | 4834 | 688.16 | 14532 | ^5^D_4_→^7^F_0_ | 5950 |
| 811.7 | 12320 |  |  |  | 4913 |  |  |  |  |
| 817.36 | 12235 |  |  |  | 4998 |  |  |  |  |

Table S2. 10 K emission spectrum of **cycEu-phTb** (Fig. 3b). It is not possible to assign some emission bands to Eu^3+^ or Tb^3+^ so that both alternatives are listed in those cases.

| Wavelength | Energy (cm^-1^) | Tb^3+^ transition | Tb^3+^ CF level (cm^-1^) | Eu^3+^ transition | Eu^3+^ CF level (cm^-1^) |
| --- | --- | --- | --- | --- | --- |
| 488.83 | 20457 | ^5^D_4_→^7^F_6_ | 20457/0 |  |  |
| 493.62 b | 20258 |  | 199 |  |  |
| 542.17 sh | 18444 | ^5^D_4_→^7^F_5_ | 2013 |  |  |
| 543.08 | 18413 |  | 2044 |  |  |
| 543.91 | 18385 |  | 2072 |  |  |
| 544.78 | 18356 |  | 2101 |  |  |
| 545.74 sh | 18324 |  | 2133 |  |  |
| 547.56 b | 18263 |  | 2194 |  |  |
| 549.81 | 18188 |  | 2269 |  |  |
| 580.17 | 17236 |  |  |  | 17236/0 |
| 583.36 | 17142 | ^5^D_4_ → ^7^F_4_ | 3315 |  | 94 |
| 587.74 | 17014 |  | 3443 | ^5^D_0_ →^7^F_1_ | 222 |
| 589.5 sh | 16964 |  | 3493 |  | 272 |
| 591.52 | 16906 |  | 3551 |  | 331 |
| 592.71 | 16872 |  | 3585 |  | 365 |
| 593.91 | 16838 |  | 3619 |  | 399 |
| 595.6 sh | 16790 |  | 3667 |  | 446 |
| 611.98 | 16340 |  | 4117 | ^5^D_0_ →^7^F_2_ | 896 |
| 613.77 | 16293 |  | 4164 |  | 944 |
| 615.21 | 16255 |  | 4202 |  | 982 |
| 616.24 | 16227 |  | 4230 |  | 1009 |
| 618.68 | 16163 |  | 4294 |  | 1073 |
| 622.31 | 16069 | ^5^D_4_ → ^7^F_3_ | 4388 |  | 1167 |
| 624.3 sh | 16018 |  | 4439 |  | 1218 |
| 628.67 | 15907 |  | 4550 |  | 1330 |
| 630.55 | 15859 |  | 4598 |  | 1377 |
| 646.41 | 15470 |  | 4987 |  | 1766 |
| 649.84 | 15388 |  | 5069 | ^5^D_0_ →^7^F_3_ | 1848 |
| 650.73 | 15367 |  | 5090 |  | 1869 |
| 653.60 | 15300 | ^5^D_4_ → ^7^F_2_ | 5157 |  | 1936 |
| 665.94 | 15016 |  | 5441 |  | 2220 |
| 668.91 | 14950 | ^5^D_4_ → ^7^F_1_ | 5507 |  | 2287 |
| 674.61 | 14823 |  | 5634 |  | 2413 |
| 677.70 | 14756 |  | 5701 |  | 2481 |
| 681.27 sh | 14678 |  | 5779 |  | 2558 |
| 682.10 | 14661 |  | 5796 |  | 2576 |
| 685.32 | 14592 |  | 5865 | ^5^D_0_ →^7^F_4_ | 2645 |
| 688.29 | 14529 | ^5^D_4_ → ^7^F_0_ | 5928 |  | 2708 |
| 690.07 | 14491 |  |  |  | 2745 |
| 691.47 b | 14462 |  |  |  | 2774 |
| 695.95 b | 14369 |  |  |  | 2597 |
| 697.40 | 14339 |  |  |  | 2897 |
| 701.00 | 14265 |  |  |  | 2971 |
| 704.96 | 14185 |  |  |  | 3051 |
| 706.58 | 14153 |  |  |  | 3084 |
| 741.36 | 13489 |  |  | ^5^D_0_ →^7^F_5_ | 3748 |
| 751.59 | 13305 |  |  |  | 3931 |
| 759.03 | 13175 |  |  |  | 4062 |
| 762.07 | 13122 |  |  |  | 4114 |
| 810.59 | 12337 |  |  | ^5^D_0_ →^7^F_6_ | 4900 |
| 812.61 | 12306 |  |  |  | 4930 |
| 819.66 | 12200 |  |  |  | 5036 |
| 829.5 b | 12055 |  |  |  | 5181 |
| 843.50 | 11855 |  |  |  | 5381 |

**Decay curve fits**

Fits were made to ^5^D_0_ → ^7^F_4_ Eu^3+^ and ^5^D_4_ →^7^F_5_ Tb^3+^ emission decay at different temperatures using the monoexponential equation:

*y* = *y*_0_ + A1*exp(-*x*/τ)

and biexponential equation:

*y* = *y*_0_ + A1*exp(-*x*/τ_1_) + A2*exp(-*x*/τ_2_)

where *y* is emission counts and *x* is time (s). In each case, *R*_adj_^2^ is the adjusted coefficient of determination. The monoexponential fits in blue font start at ~0.2 ms after the laser pulse. Excitation wavelength into antenna: 355 nm; emission wavelengths 543-546 nm (Tb^3+^) and 700 nm (Eu^3+^). The tabulated values of τ_1_ and τ_2_ below are in s.

The decay curves were also integrated to obtain the “steady-state” lifetime, $\tau_{\mathrm{ST}}=\int_{0}^{\infty} \frac{I(t)}{I(0)}dt$. Now *k*’’_ET_ = (1/τ_0_)[(*η*_0_/*η*)-1] where τ_0_ is the Tb^3+^ lifetime in the absence of Eu^3+^; *η*_0_/*η* is the ratio of the quantum efficiency of Tb^3+^ emission in the absence of energy transfer to the quantum efficiency of Tb^3+^ emission in the presence of energy transfer. But $\frac{\eta}{\eta_{0}}=\frac{1}{\tau_{0}}\int_{0}^{\infty} I\left( t \right)dt$ where *I*(0) is normalised to 1.

Table S3. Decay curve fits for **cycTb-phEu**: Eu^3+^ emission.

| Temp K | Monoexp: *y*_0_ | A1 | τ | *R*_adj_^2^ | Biexp: A1 | τ_1_ | A2 | τ_2_ | *R*_adj_^2^ | τ_ST_ |
| --- | --- | --- | --- | --- | --- | --- | --- | --- | --- | --- |
| 10 | 0.0003 | 0.02789 | 2.77E-4 | 0.99158 | 0.01855 | 3.77E-4 | 0.01441 | 9.64E-5 | 0.99736 | 2.04E-4 |
|  | 2.44E-4 | 0.0221 | 3.36E-4 | 0.99307 |  |  |  |  |  |  |
| 50 | 0.00053 | 0.10332 | 2.92E-4 | 0.99553 | 0.06898 | 3.85E-4 | 0.0483 | 1.16E-4 | 0.99917 | 1.85E-4 |
|  | 5.62E-4 | 0.08685 | 3.34E-4 | 0.99732 |  |  |  |  |  |  |
| 100 | 0.00057 | 0.1109 | 2.94E-4 | 0.99617 | 0.07493 | 3.83E-4 | 0.04773 | 1.21E-4 | 0.99929 | 1.60E-4 |
|  | 6.22E-4 | 0.09445 | 3.33E-4 | 0.99758 |  |  |  |  |  |  |
| 150 | 0.00067 | 0.15203 | 2.82E-4 | 0.9968 | 0.11707 | 3.42E-4 | 0.04913 | 9.65E-5 | 0.99935 | 1.24E-4 |
|  | 6.74E-4 | 0.13119 | 3.17E-4 | 0.99805 |  |  |  |  |  |  |
| 200 | 0.00042 | 0.09915 | 2.56E-4 | 0.99641 | 0.063 | 3.36E-4 | 0.0483 | 1.17E-4 | 0.99908 | 6.8E-5 |
|  | 4.34E-4 | 0.08361 | 2.90E-4 | 0.99698 |  |  |  |  |  |  |
| 250 | 0.00016 | 0.05085 | 2.02E-4 | 0.99231 | 0.03215 | 2.81E-4 | 0.02676 | 7.38E-5 | 0.99907 | 4.0E-5 |
|  | 1.46E-4 | 0.03646 | 2.61E-4 | 0.99631 |  |  |  |  |  |  |
| 298 | 0.00033 | 0.07071 | 1.74E-4 | 0.98781 | 0.06099 | 1.98E-4 | 0.09805 | 1.87E-5 | 0.99553 | 3.0E-5 |
|  | 2.45E-4 | 0.04292 | 2.51E-4 | 0.99271 |  |  |  |  |  |  |

Table S4. Decay curve fits for **cycTb-phEu**: Tb^3+^ emission.

| Temp K | Monoexp: *y*_0_ | A1 | τ | *R*_adj_^2^ | biexp: A1 | τ_1_ | A2 | τ_2_ | *R*_adj_^2^ | τ_ST_ |
| --- | --- | --- | --- | --- | --- | --- | --- | --- | --- | --- |
| 10 | 5.019E-4 | 0.04913 | 1.64E-4 | 0.9156 | 0.01714 | 5.00E-4 | 0.04201 | 5.99E-5 | 0.99516 | 2.67E-5 |
|  | 5.287E-4 | 0.01926 | 4.17E-4 | 0.98561 |  |  |  |  |  |  |
| 50 | 4.21E-4 | 0.04007 | 2.911E-4 | 0.97089 | 1.204E-4 | 5.79E-4 | 0.03987 | 9.72E-5 | 0.99719 | 3.17E-5 |
|  | 6.31E-4 | 0.02567 | 4.13E-4 | 0.9883 |  |  |  |  |  |  |
| 100 | 3.37E-4 | 0.03143 | 2.31E-4 | 0.96884 | 0.01285 | 4.84E-4 | 0.03363 | 8.15E-5 | 0.99598 | 2.67E-5 |
|  | 3.95E-4 | 0.01781 | 3.65E-4 | 0.98344 |  |  |  |  |  |  |
| 150 | 2.652E-4 | 0.02814 | 1.88E-4 | 0.96853 | 0.01085 | 4.035E-4 | 0.03189 | 6.889E-5 | 0.995 | 1.77E-5 |
|  | 2.363E-4 | 0.01417 | 3.24E-4 | 0.97909 |  |  |  |  |  |  |
| 200 | 8.825E-5 | 0.02011 | 8.599E-5 | 0.94389 | 0.00952 | 1.775E-4 | 0.03183 | 1.731E-5 | 0.99197 | 8.1E-6 |
|  | 1.264E-5 | .00532 | 2.6666E-4 | 0.96161 |  |  |  |  |  |  |
| 250 | 6.75E-5 | 0.02646 | 4.256E-5 | 0.91875 | 0.0098 | 1.122E-4 | 0.0539 | 9.420E-6 | 0.99265 | 7.0E-6 |
|  | 1.56E-5 | 0.00511 | 1.78E-4 | 0.96152 |  |  |  |  |  |  |
| 298 | 5.33E-6 | 0.07023 | 5.978E-6 | 0.95595 | 0.00425 | 4.998E-5 | 0.08629 | 4.237E-6 | 0.99131 | 5.4E-6 |
| 298 refit | 1.008E-5 | 0.02643 | 1.166E-5 | 0.92012 |  |  |  |  |  |  |
|  | -2.50E-5 | 0.00131 | 1.135E-4 | 0.80106 |  |  |  |  |  |  |

Table S5. Decay curve fits for **cycEu-phTb**: Eu^3+^ emission.

| Temp K | Monoexp: *y*_0_ | A1 | τ | *R*_adj_^2^ | Biexp: A1 | τ_1_ | A2 | τ_2_ | *R*_adj_^2^ | τ_ST_ |
| --- | --- | --- | --- | --- | --- | --- | --- | --- | --- | --- |
| 10 | 2.326E-4 | 0.04748 | 6.355E-4 | 0.99782 | 0.01003 | 2.394E-4 | 0.03977 | 7.262E-4 | 0.99874 | 4.23E-4 |
|  | 4.893E-4 | 0.04579 | 6.431E-4 | 0.99736 |  |  |  |  |  |  |
| 50 | 2.537E-4 | 0.04411 | 6.423E-4 | 0.99786 | 0.01144 | 2.989E-4 | .0346 | 7.536E-4 | 0.9986 | 4.25E-4 |
|  | 4.841E-4 | 0.04276 | 6.471E-4 | 0.99704 |  |  |  |  |  |  |
| 100 | 2.517E-4 | 0.04243 | 6.493E-4 | 0.99812 | 0.00955 | 3.055E-4 | 0.03445 | 7.461E-4 | 0.99861 | 3.92E-4 |
|  | 3.887E-4 | 0.04133 | 6.566E-4 | 0.99729 |  |  |  |  |  |  |
| 150 | 6.694E-4 | 0.09959 | 6.099E-4 | 0.99637 | 0.09688 | 6.302E-4 | 0.03797 | 1.464E-5 | 0.99849 | 3.61E-4 |
| Refit 150 | 5.807E-4 | 0.09636 | 6.322E-4 | 0.99839 | 0.0264 | 3.050E-4 | 0.0751 | 7.352E-4 | 0.99898 |  |
|  | 0.00106 | 0.09431 | 6.316E-4 | 0.99805 |  |  |  |  |  |  |
| 200 | 6.987E-5 | 0.0207 | 6.307E-4 | 0.99686 | 0.01855 | 6.895E-4 | 0.00307 | 1.991E-4 | 0.99745 | 3.44E-4 |
|  | 1.399E-4 | 0.02008 | 6.398E-4 | 0.99487 |  |  |  |  |  |  |
| 250 | 3.861E-4 | 0.08085 | 5.8117E-4 | 0.99794 | 0.06835 | 6.594E-4 | 0.01848 | 2.0123E-4 | 0.99892 | 3.08E-4 |
|  | 6.621E-4 | 0.07776 | 5.928E-4 | 0.99785 |  |  |  |  |  |  |
| 298 | 4.622E-4 | 0.08197 | 5.626E-4 | 0.99768 | 0.0705 | 6.370E-4 | 0.0182 | 1.648E-4 | 0.999 | 2.41E-4 |
|  | 6.723E-5 | 0.07773 | 5.833E-4 | 0.99786 |  |  |  |  |  |  |

Table S6. Decay curve fits for **cycEu-phTb**: Tb^3+^ emission.

| Temp K | Monoexp: *y*_0_ | A1 | Τ | *R*_adj_^2^ | Biexp: A1 | τ_1_ | A2 | τ_2_ | *R*_adj_^2^ | τ_ST_ |
| --- | --- | --- | --- | --- | --- | --- | --- | --- | --- | --- |
| 10 | 7.795E-4 | 0.03767 | 5.081E-4 | 0.97312 | 0.02672 | 7.343E-4 | 0.03138 | 8.148E-5 | 0.99679 | 3.11E-4 |
|  | 9.675E-4 | 0.0295 | 6.223E-4 | 0.99231 |  |  |  |  |  |  |
| 50 | 4.464E-4 | 0.02069 | 4.053E-4 | 0.95636 | 0.0131 | 6.734E-4 | 0.01903 | 6.640E-5 | 0.99665 | 2.98E-4 |
|  | 4.634E-4 | 0.014099 | 5.833E-4 | 0.99056 |  |  |  |  |  |  |
| 100 | 3.470E-4 | 0.01174 | 1.2646E-4 | 0.8896 | 0.0048 | 3.730E-4 | 0.01825 | 2.505E-5 | 0.98714 | 1.50E-4 |
|  | 9.842E-5 | 0.00383 | 4.606E-4 | 0.95608 |  |  |  |  |  |  |
| 150 | 1.456E-4 | 0.01376 | 1.584E-4 | 0.94131 | 0.00528 | 3.934E-4 | 0.01901 | 4.602E-5 | 0.99128 | 9.3E-5 |
|  | 4.887E-5 | 0.00529 | 3.801E-4 | 0.96429 |  |  |  |  |  |  |
| 200 | 1.015E-4 | 0.03767 | 1.472E-5 | 0.91262 | 0.00394 | 1.602E-4 | 0.04374 | 8.863E-6 | 0.98963 | 4.9E-5 |
|  | 5.344E-5 | 0.00298 | 1.940E-4 | 0.93779 |  |  |  |  |  |  |

Table S7. Decay curve fits for **cycTb-phen**: Tb^3+^ emission.

| Temp K | Monoexp: *y*_0_ | A1 | τ | *R*_adj_^2^ | Biexp: A1 | τ_1_ | A2 | τ_2_ | *R*_adj_^2^ | τ_ST_ |
| --- | --- | --- | --- | --- | --- | --- | --- | --- | --- | --- |
| 10 | 0.0013 | 0.02514 | 5.341E-4 | 0.98664 | 0.01941 | 7.489E-4 | 0.01054 | 1.255E-4 | 0.99547 | 5.14E-4 |
|  | 8.34E-4 | 0.0221 | 6.51E-4 | 0.99111 |  |  |  |  |  |  |
| 50 | 0.00126 | 0.02414 | 4.842E-4 | 0.98527 | 0.01082 | 1.0377E-4 | 0.01853 | 6.893E-4 | 0.99627 | 4.20E-4 |
|  | 7.19E-4 | 0.02061 | 6.172E-4 | 0.99202 |  |  |  |  |  |  |
| 100 | 9.69E-4 | 0.02129 | 3.941E-4 | 0.98383 | 0.01117 | 1.047E-4 | 0.01488 | 5.976E-4 | 0.99554 | 3.01E-4 |
|  | 4.97E-4 | 0.01741 | 5.22E-4 | 0.98951 |  |  |  |  |  |  |
| 150 | 6.39E-4 | 0.02067 | 2.941E-4 | 0.98204 | 0.01301 | 4.716E-4 | 0.01227 | 8.553E-5 | 0.99716 | 2.19E-4 |
|  | 3.04E-4 | 0.01526 | 4.18E-4 | 0.99116 |  |  |  |  |  |  |
| 200 | 5.042E-4 | 0.02843 | 1.922E-4 | 0.98187 | 0.01488 | 3.361E-4 | 0.01965 | 7.064E-5 | 0.99674 | 1.58E-4 |
|  | 2.00E-4 | 0.01819 | 2.96E-4 | 0.98773 |  |  |  |  |  |  |
| 250 | 3.667E-4 | 0.03443 | 1.334E-4 | 0.98466 | 0.01596 | 2.371E-4 | 0.02693 | 5.573E-5 | 0.99724 | 9.8E-5 |
|  | 1.38E-4 | 0.01939 | 2.14E-4 | 0.98761 |  |  |  |  |  |  |
| 298 | 1.301E-4 | 0.0331 | 4.245E-5 | 0.97681 | 0.03076 | 1.806E-5 | 0.01235 | 8.950E-5 | 0.99622 | 3.9E-5 |
|  | 3.76E-6 | 0.00605 | 1.33E-4 | 0.94305 |  |  |  |  |  |  |


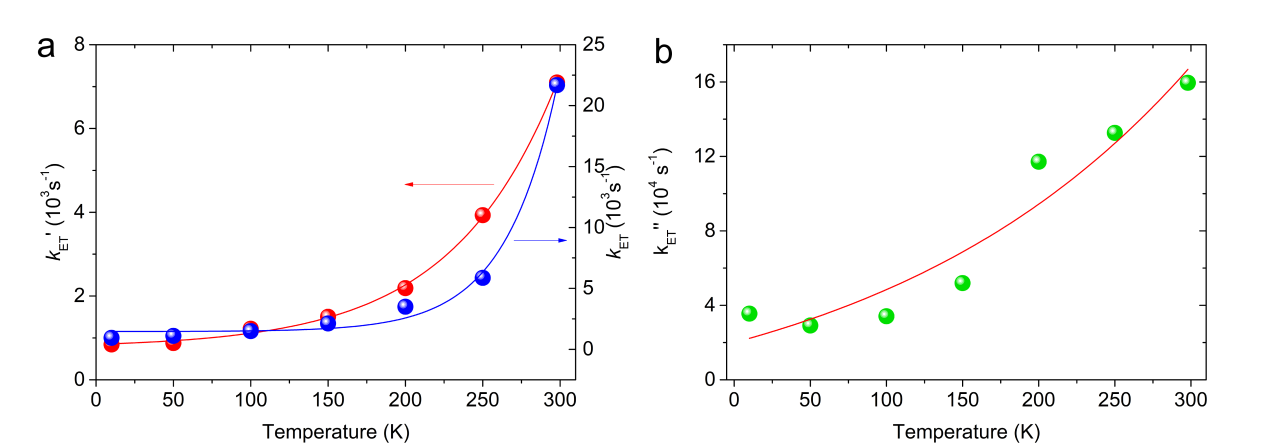


Figure S8. (a) Exponential growth models for Tb^3+^ → Eu^3+^ energy transfer rates deduced from long-term lifetimes (*k*_ET_’: black datapoints, red fitted curve) and subtracted decay curves (*k*_ET:_ blue datapoints, blue fitted curve). The fitting equations are:

*k*_ET_’ = (768±58) + (79±13)exp[(68±2)/*T*], *R*_adj_^2^ = 0.9989

*k*_ET_ = (1456±330) + (3.1±2.7)exp[(34±3)/*T*], *R*_adj_^2^ = 0.9925

(b) Exponential growth model for Tb^3+^ → Eu^3+^ energy transfer rates deduced from steady-state lifetimes with the fitting equation:

*k*_ET_’’ = (-2.6±7.8)x10^4^ + (4.6±6.6)x10^4^exp[(-208±156)/*T*], *R*_adj_^2^ = 0.8936

Table S8. Biexponential fits, *y* = A1*exp(-*x*/τ_1_) + A2*exp(-*x*/τ_2_) + *y*_0_, of subtracted decay curves of **cycTb-phEu** and **cycTb-phen** at different temperatures.

| Temp K | *y*_0_ | A1 | τ_1_ | A2 | τ_2_ | Radj^2^ |
| --- | --- | --- | --- | --- | --- | --- |
| 10 | 0.0088 | 0.34493 | 0.00106 | -0.4109 | 3.119E-5 | 0.98019 |
| 50 | 0.0032 | 0.28029 | 9.07E-4 | -0.41417 | 3.589E-5 | 0.9789 |
| 100 | 0.0057 | 0.28008 | 6.698E-4 | -0.47695 | 2.007E-5 | 0.98064 |
| 150 | 0.0056 | 0.28162 | 4.721E-4 | -0.51781 | 1.542E-5 | 0.98478 |
| 200 | 0.0044 | 0.26531 | 2.862E-4 | -0.54833 | 1.183E-5 | 0.97128 |
| 250 | 0.0031 | 0.28098 | 1.707E-4 | -0.61174 | 1.123E-5 | 0.94877 |
| 298 | 0.0014 | 0.31275 | 4.613E-5 | -0.72922 | 8.074E-6 | 0.81566 |

Table S9. Solid-state Tb^3+^ - Eu^3+^ energy transfer parameters (hfa hexafluoro acetylacetonato; dpbp 4,4’-bis(diphenylphosphoryl)biphenyl; oxy oxydiacetate).

| System | T (K) | Closest cation distance (Å) | Cation C.N. | λ_exc_ (nm) | η(ET) % | ET rate (ms)^-1^ | Ref. |
| --- | --- | --- | --- | --- | --- | --- | --- |
| cycTb-phEu | 298 | 10.6 | 8 | 355 | 81 | 7.1 | This work |
|  | 10 |  |  | 355 | 35 | 0.84 | This work |
| [Tb_0.99_Eu_0.01_(hfa)_3_ (dpbp)]_n_ | 200 | I |  | 355 |  | 1 | [1](#_ENREF_1) |
| [Tb_0.99_Eu_0.01_(hfa)_3_ (dpbp)]_n_ | 300 |  |  | 355 | 38 |  | [1](#_ENREF_1) |
| Y_0.46_Ga_1.5_Al_1.5_(BO_3_)_4_:Tb_0.50_Eu_0.04_ | 300 | 7.1 | 6 | 374 | 38 | 0.32 | [2](#_ENREF_2) |
| Y_0.34_Ga_1.5_Al_1.5_(BO_3_)_4_:Tb_0.50_Eu_0.16_ | 300 |  | 6 | 374 | 74 | 1.50 | [2](#_ENREF_2) |
| Cs_2_NaTb_0.99_Eu_0.01_Cl_6_ | 293 | 7.6 | 6 | 488 | 9 | 0.015 | [3](#_ENREF_3) |
|  | 80 |  |  | 488 | 5 | 0.006 | [3](#_ENREF_3) |
| Cs_2_NaTb_0.85_Eu_0.15_Cl_6_ | 293 | 7.6 | 6 | 488 | 87 | 1.01 | [3](#_ENREF_3) |
|  | 80 |  |  | 488 | 77 | 0.35 | [3](#_ENREF_3) |
| ([(Eu_0.5_Tb_0.5_)(C_6_H_8_O_4_)_3_- (H_2_O)_2_] (C_10_H_8_N_2_) | 295 | 4.053 | 9 | 285 | 45 | 0.59 | [4](#_ENREF_4) |
| Na_3_[Tb_0.01_Eu_0.99_(oxy)_3_].2NaC1O_4_.6H_2_O | 298 | 9.4 | 9 | 488 | 23 | 0.11 | [5](#_ENREF_5) |
|  | 77 |  |  | 488 | 0.4 | 0.0017 | [5](#_ENREF_5) |
| Sr_3_Tb_0.90_Eu_0.10_(PO_4_)_3_ | 298 | 4 | 6 | 355 | 93 | 1.67 | [6](#_ENREF_6) |
| Ca_3_Tb_1.90_Eu_0.10_Si_3_O_12_ | 300 | Est 7.94 |  |  | 0.94 | 94 | [7](#_ENREF_7) |

Table S10. Comparison of bond distances (in Å) from optimizations of (a) 166-atom optimization (Fig. S37) using RM1 in MOPAC; (b) 100-atom optimization of simplified structure (Figure 1c) using PBeh-3c in ORCA.

| Bond distance | MOPAC^a^ | PBeh-3c^b^ |
| --- | --- | --- |
| Eu-S | 2.85±0.06 | 2.92±0.01 |
| Eu-N | 2.51±0.09 | 2.56±0.00 |
| Tb-O | 2.30±0.05 | 2.30±0.04 |
| Tb-N | 2.40±0.11 | 2.41±0.07 |
| Tb-Eu | 10.46 | 10.69 |

Table S11. Room temperature overall energy transfer rates (s^-1^) (= forward transfer rate – backward transfer rate) to Eu^3+^ levels from excited ligand singlet states.

1. Calculation 1: **cycEu-phTb**

| Singlet state energy  (cm^-1^) | Transfer rate to Eu^3+^ *J*-multiplet | | | | |
| --- | --- | --- | --- | --- | --- |
|  | ^5^D_4_ | ^5^G_6_ | ^5^L_6_ | ^5^D_1_ | ^5^D_0_ |
| 23084 | -1.2E9 | -1.5E6 | -1.1E4 | 4.0E-7 | 2.5E-7 |
| 24059 | -1.8E7 | -2.0E4 | -1.3E2 | 2.2E-7 | 1.1E-7 |
| 25556 | -2.4E4 | -2.3E1 | 2.3E-1 | 7.1E-8 | 2.5E-8 |
| 25921 | -1.4E4 | -6.4E0 | 4.9E-1 | 1.9E-7 | 6.3E-8 |
| 26837 | -4.9E2 | 8.0E-2 | 8.4E-1 | 4.7E-7 | 1.3E-7 |
| 27226 | -2.0E1 | 1.1E-1 | 4.1E-1 | 5.0E-8 | 1.2E-8 |
| 27434 | -5.6E0 | 1.3E-1 | 4.3E-1 | 5.3E-8 | 1.2E-8 |
| 27782 | 2.9E0 | 1.2E-1 | 3.7E-1 | 3.1E-8 | 6.7E-9 |
| 28550 | 3.9E0 | 9.9E-2 | 2.6E-1 | 1.0E-8 | 1.8E-9 |

(b) Calculation 2: **cycEu-phTb**

| Singlet state energy  (cm^-1^) | Transfer rate to Eu^3+^ *J*-multiplet | | | | |
| --- | --- | --- | --- | --- | --- |
|  | ^5^D_4_ | ^5^G_6_ | ^5^L_6_ | ^5^D_1_ | ^5^D_0_ |
| 23084 | -1.2E9 | -1.5E6 | -1.1E4 | 4.0E-7 | 2.5E-7 |
| 24059 | -1.8E7 | -2.0E4 | -1.3E2 | 2.2E-7 | 1.1E-7 |
| 25556 | -2.4E4 | -2.3E1 | 2.3E-1 | 7.1E-8 | 2.5E-8 |
| 25921 | -1.4E4 | -6.4E0 | 4.9E-1 | 1.9E-7 | 6.3E-8 |
| 26837 | -4.9E2 | 8.0E-2 | 8.4E-1 | 4.7E-7 | 1.3E-7 |
| 27226 | -2.0E1 | 1.1E-1 | 4.1E-1 | 5.0E-8 | 1.2E-8 |
| 27434 | -5.6E0 | 1.3E-1 | 4.3E-1 | 5.3E-8 | 1.2E-8 |
| 27782 | 2.9E0 | 1.2E-1 | 3.7E-1 | 3.1E-8 | 6.7E-9 |
| 28550 | 3.9E0 | 9.9E-2 | 2.6E-1 | 1.0E-8 | 1.8E-9 |

(c) Calculation 3: **cycTb-phEu**

| Singlet state energy  (cm^-1^) | Transfer rate to Eu^3+^ *J*-multiplet | | | | |
| --- | --- | --- | --- | --- | --- |
|  | ^5^D_4_ | ^5^G_6_ | ^5^L_6_ | ^5^D_1_ | ^5^D_0_ |
| 23059 | -1.3E9 | -1.6E6 | -1.3E4 | 4.0E-7 | 2.5E-7 |
| 24044 | -1.9E7 | -2.2E4 | -1.4E2 | 2.2E-7 | 1.1E-7 |
| 25497 | -3.1E4 | -3.0E1 | 1.9E-1 | 7.5E-8 | 2.7E-8 |
| 25889 | -1.3E4 | -7.5E0 | 4.8E-1 | 2.0E-7 | 6.5E-8 |
| 26766 | -6.9E2 | 1.5E-2 | 8.6E-1 | 5.1E-7 | 1.4E-7 |
| 27121 | -3.5E1 | 1.0E-1 | 4.2E-1 | 5.6E-8 | 1.4E-8 |
| 27186 | -1.9E1 | 9.4E-2 | 3.6E-1 | 3.4E-8 | 8.5E-9 |
| 27421 | -6.2E0 | 1.3E-1 | 4.3E-1 | 5.4E-8 | 1.3E-8 |
| 27704 | 2.0E0 | 1.2E-1 | 3.8E-1 | 3.4E-8 | 7.4E-9 |
| 28509 | 3.9E0 | 1.0E-1 | 2.6E-1 | 1.1E-8 | 2.0E-9 |
| 29396 | 3.5E0 | 8.0E-2 | 1.8E-1 | 3.6E-9 | 5.4E-10 |
| 29533 | 4.1E0 | 8.4E-2 | 1.8E-1 | 4.0E-9 | 5.8E-10 |

Table S12. Room temperature overall energy transfer rates (s^-1^) (= forward transfer rate – backward transfer rate) to Eu^3+^ levels from excited triplet states.

(a) Calculation 1: **cycEu-phTb**

| Triplet state energy  (cm^-1^) | Transfer rate to Eu^3+^ *J*-multiplet | | | | |
| --- | --- | --- | --- | --- | --- |
|  | ^5^D_4_ | ^5^G_6_ | ^5^L_6_ | ^5^D_1_ | ^5^D_0_ |
| 20501 | -2.7E14 | -1.1E11 | -1.4E9 | 9.6E-5 | 1.1E-4 |
| 23015 | -5.2E8 | -6.6E5 | -5.1E3 | 4.1E-7 | 2.6E-7 |
| 23981 | -4.7E7 | -2.3E4 | -1.5E2 | 3.0E-6 | 1.6E-6 |
| 24239 | -2.6E7 | -9.9E3 | -6.1E1 | 5.7E-6 | 2.8E-6 |
| 25172 | -1.1E5 | -7.1E1 | -1.9E-1 | 3.8E-7 | 1.5E-7 |
| 25296 | -1.8E5 | -7.0E1 | -4.7E-2 | 1.7E-6 | 6.4E-7 |
| 25920 | -1.5E4 | -4.6E0 | 3.4E-1 | 1.7E-6 | 5.5E-7 |
| 26451 | -1.6E3 | -3.3E-1 | 3.9E-1 | 1.4E-6 | 4.2E-7 |
| 26536 | -2.0E2 | -7.6E-2 | 1.6E-1 | 1.0E-7 | 2.9E-8 |
| 26823 | -3.2E2 | 3.1E-2 | 3.9E-1 | 1.2E-6 | 3.1E-7 |
| 27411 | -2.3E0 | 4.3E-2 | 1.4E-1 | 5.5E-8 | 1.3E-8 |
| 27656 | 4.5E-1 | 4.1E-2 | 1.3E-1 | 3.6E-8 | 7.9E-9 |
| 27864 | 1.5E0 | 4.7E-2 | 1.4E-1 | 4.4E-8 | 9.2E-9 |
| 28412 | 1.8E0 | 4.0E-2 | 1.1E-1 | 2.0E-8 | 3.7E-9 |

(b) Calculation 2: **cycEu-phTb**

| Triplet state energy  (cm^-1^) | Transfer rate to Eu^3+^ *J*-multiplet (s^-1^) | | | | |
| --- | --- | --- | --- | --- | --- |
|  | ^5^D_4_ | ^5^G_6_ | ^5^L_6_ | ^5^D_1_ | ^5^D_0_ |
| 18946 | -2.3E17 | -9.2E16 | -1.5E15 | -1.1E-4 | 3.5E-4 |
| 20946 | -3.5E15 | -1.3E13 | -1.5E11 | 7.7E-6 | 7.8E-6 |
| 21918 | -7.1E11 | -2.5E11 | -2.3E9 | 5.6E-6 | 4.6E-6 |
| 23324 | -3.1E9 | -8.8E8 | -6.4E6 | 8.2E-6 | 4.8E-6 |
| 23419 | -2.8E9 | -7.5E8 | -5.4E6 | 1.7E-5 | 9.6E-6 |
| 23491 | -1.8E9 | -4.9E8 | -3.5E6 | 1.2E-5 | 6.7E-6 |
| 24326 | -6.5E7 | -1.5E7 | -9.3E4 | 1.4E-5 | 6.5E-6 |
| 24869 | -3.7E6 | -8.8E5 | -4.3E3 | 2.7E-6 | 1.1E-6 |
| 25230 | -1.0E6 | -2.3E5 | -4.3E2 | 4.9E-6 | 1.9E-6 |
| 25485 | -1.8E5 | -4.2E4 | 2.4E2 | 8.3E-7 | 3.0E-7 |
| 25768 | -9.2E4 | -1.9E4 | 6.5E2 | 3.1E-6 | 1.1E-6 |
| 26117 | -2.4E4 | -4.4E3 | 8.7E3 | 4.4E-6 | 1.4E-6 |
| 26507 | -2.1E3 | -2.9E2 | 4.9E2 | 6.1E-7 | 1.8E-7 |
| 26634 | -2.3E3 | -1.8E2 | 8.6E2 | 3.0E-6 | 8.4E-7 |
| 27001 | -2.8E2 | 1.2E2 | 6.1E2 | 9.0E-7 | 2.3E-7 |
| 27071 | -1.4E2 | 1.0E2 | 4.5E2 | 3.6E-7 | 9.2E-8 |
| 27394 | -2.0E1 | 1.2E2 | 4.1E2 | 2.5E-7 | 5.8E-8 |
| 27572 | -8.0E-1 | 1.0E2 | 3.2E2 | 1.1E-7 | 2.6E-8 |
| 27944 | 1.0E1 | 1.2E2 | 3.4E2 | 1.2E-7 | 2.5E-8 |
| 28083 | 8.2E0 | 8.6E1 | 2.4E2 | 4.5E-8 | 9.0E-9 |

(c) Calculation 3: **cycTb-phEu**

| Triplet state energy  (cm^-1^) | Transfer rate to Eu^3+^ *J*-multiplet | | | | |
| --- | --- | --- | --- | --- | --- |
|  | ^5^D_4_ | ^5^G_6_ | ^5^L_6_ | ^5^D_1_ | ^5^D_0_ |
| 19597 | -7.7E15 | -3.6E12 | -5.3E10 | 8.9E-5 | 1.3E-4 |
| 22971 | -6.3E8 | -8.0E5 | -6.2E3 | 4.2E-7 | 2.7E-7 |
| 23932 | -5.8E7 | -2.9E4 | -1.9E2 | 3.1E-6 | 1.6E-6 |
| 24127 | -4.0E7 | -1.6E4 | -9.9E1 | 5.6E-6 | 2.8E-6 |
| 24889 | -8.4E5 | -3.9E2 | -1.9E0 | 1.5E-6 | 6.2E-7 |
| 25134 | -1.3E5 | -8.5E1 | -2.7E-1 | 3.9E-7 | 1.5E-7 |
| 25710 | -3.3E4 | -1.1E1 | 2.8E-1 | 1.5E-6 | 5.2E-7 |
| 26273 | -4.9E3 | -1.0E0 | 4.6E-1 | 2.5E-6 | 7.6E-7 |
| 26412 | -3.4E2 | -1.6E-1 | 1.5E-1 | 1.0E-7 | 3.0E-8 |
| 26502 | -1.6E2 | -7.8E-2 | 1.3E-1 | 5.6E-8 | 1.6E-8 |
| 26687 | -4.8E2 | -3.6E-2 | 3.5E-1 | 9.4E-7 | 2.6E-7 |
| 27356 | -3.5E0 | 4.3E-2 | 1.4E-1 | 5.8E-8 | 1.4E-8 |
| 27548 | -3.4E-1 | 4.2E-2 | 1.4E-1 | 4.5E-8 | 1.0E-8 |
| 27782 | 3.0E0 | 7.5E-2 | 2.2E-1 | 1.8E-7 | 3.8E-8 |
| 27808 | 1.4E0 | 4.7E-2 | 1.4E-1 | 4.7E-8 | 1.0E-8 |
| 28263 | 2.8E0 | 5.3E-2 | 1.5E-1 | 4.7E-8 | 9.1E-9 |


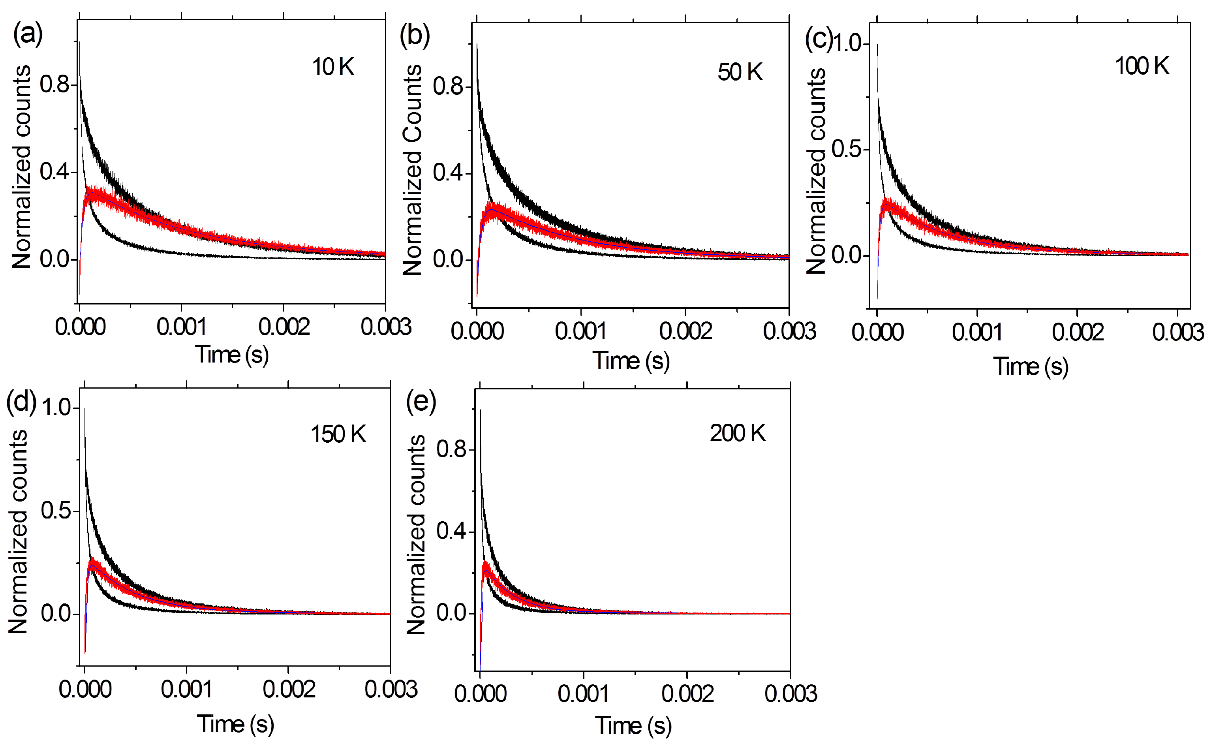


Fig. S9. Subtraction of decay curves of **cycTb-phen** and **cycTb-phEu**. The subtracted curves are in red and the fitting in blue. The subtraction using Origin was checked by manually subtracting the curves.


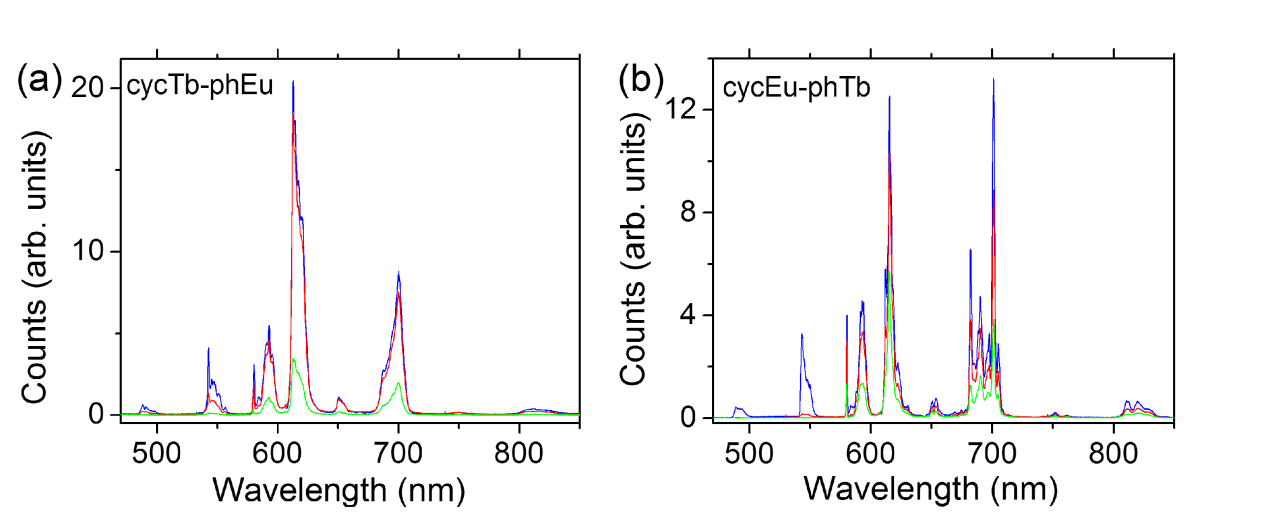


Fig. S10. The emission spectra (λ_ex_ = 355 nm) of **cycTb-phEu** (a) and **cycEu-phTb** (b) recorded at 10 K (blue), 150 K (red) and 298 K (green).


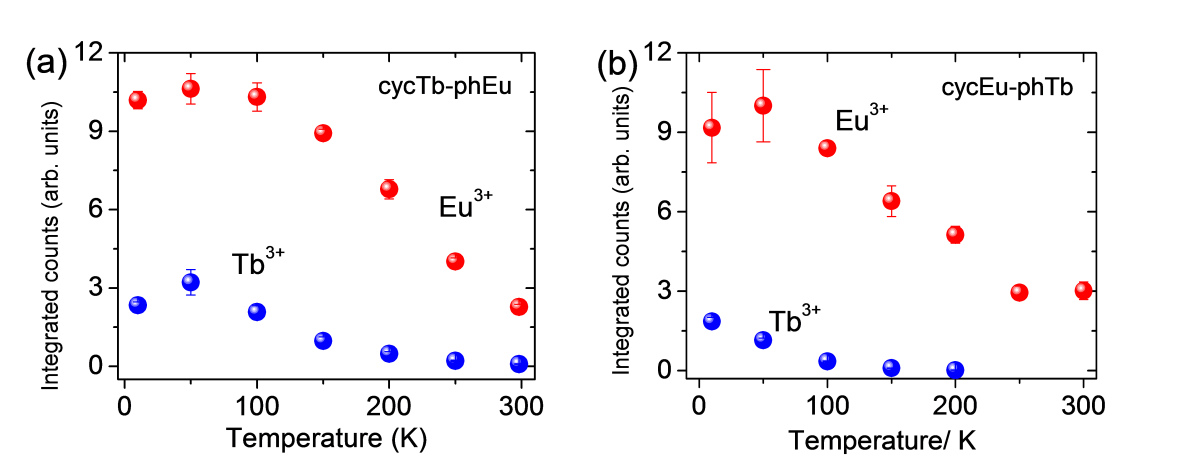


Fig. S11. The temperature-dependent integrated area of the ^5^D_0_ → ^7^F_4_ (Eu^3+^) and ^5^D_4_ → ^7^F_5_ (Tb^3+^) transitions for **cycTb-phEu** (a) and **cycEu-phTb** (b).


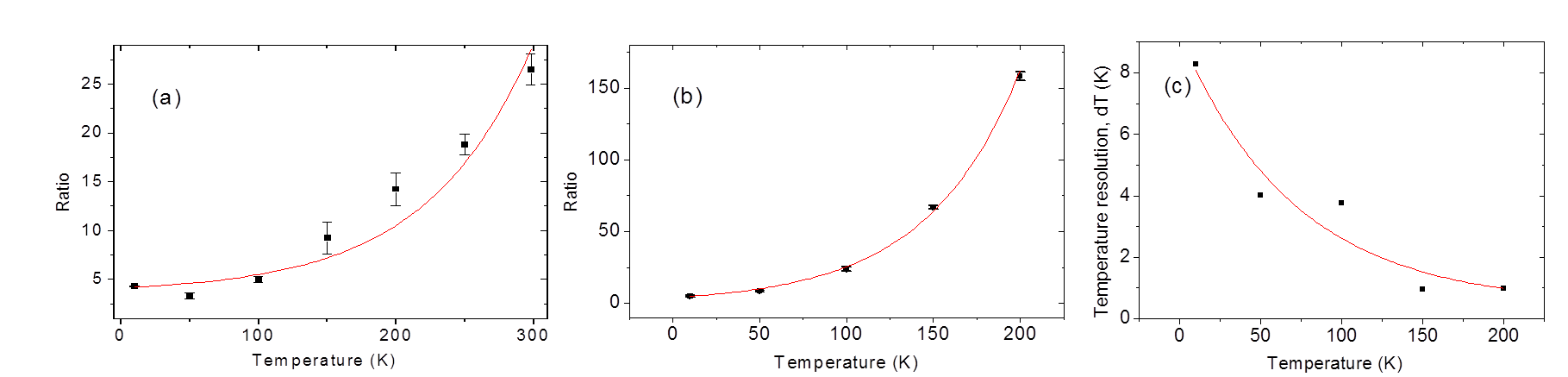


Fig. S12. Intensity ratio of ^5^D_0_ → ^7^F_4_ : ^5^D_4_ → ^7^F_5_ for (a) **cycTb-phEu** at different temperatures fitted by the exponential growth curve : *y* = -(3.7±0.6) + (0.49±0.49)exp(*x*/(76±20)), *R*_adj_^2^ = 0.8962. (b) **cycEu-phTb** at different temperatures fitted by the exponential growth curve: *y* = (3.96±0.47)exp(*x*/(53.8±2.0)), *R*_adj_^2^ = 0.9960. (c) Temperature resolution, d*T*, of **cycEu-phTb** plotted against temperature. The first order exponential decay fit is a guide to the eye.

Table S13. Literature studies of lanthanide-based thermometers and their sensitivities.

| System | Temperature range (K) | Emission wavelengths monitored (nm) | Sensitivity  (% K^-1^) | Ref |
| --- | --- | --- | --- | --- |
| [Tb_0.99_Eu_0.01_(hfa)_3_(dpbp)]_n_ | 200-300 | Not clear | 0.83 | [8](#_ENREF_8) |
| Tb_0.99_Eu_0.01_(BDC)_1.5_(H_2_O)_2_ | 300-320 | 545, 620 | 1.3 | [9](#_ENREF_9) |
| β-NaYF_4_:Yb^3+^,Er^3+^/β-  NaYF_4_:Yb^3+^,Nd^3+^ | 150-350 | 1060, 980 | 1-2 | [10](#_ENREF_10) |
| Tb_0.80_Eu_0.20_BPDA | 298-318 | 544, 614 | 0.2-1.2 | [11](#_ENREF_11) |
| Na_2_K[(Lu_0.75_Yb_0.20_Er_0.05_)_3_Si_6_O_18_] | 12-450 | 1000, 1550 | Max. 2.6 | [12](#_ENREF_12) |
| LaF_3_: Tm^3+^, Er^3+^, Yb^3+^ | 293 | 1000, 1550 | 5 | [13](#_ENREF_13) |
| [Eu,Tb (btfa)_3_(MeOH)(bpeta)]  Eu,Tb(acac).3H_2_O | 293-320 | 544, 614 | Max. 1.5 | [14](#_ENREF_14) |
| LaF_3_: Nd^3+^, Yb^3+^ | 283 | 1000, 1300 | 0.1 | [15](#_ENREF_15) |

**Calculations**

Structures were displayed in Avogadro[^16^](#_ENREF_16) and Gabedit[^17^](#_ENREF_17). Calculations were performed using MOPAC[^18^](#_ENREF_18) geometry optimizations prior to LUMPAC[^19^](#_ENREF_19)^,^[^20^](#_ENREF_20) calculation of excited singlet and triplet states. The optimized structure in MOPAC is displayed in Fig. S13.

Optimizations and energy level calculations were also carried out using ORCA[^21^](#_ENREF_21). The composite approach PBEh-3c was employed with the basis: def2-mSVP and the auxiliary basis def2/J for calculations of the 100-atom simplified structure[^22-24^](#_ENREF_22). This functional is a reparameterized version of PBE0 (with 42 % HF exchange) that uses a double-zeta basis set, def2-mSVP (unlike the minimal basis set in HF-3c) and adds 3 corrections that correct for dispersion (via D3), basis set superposition (via gCP) and other basis set incompleteness effects.


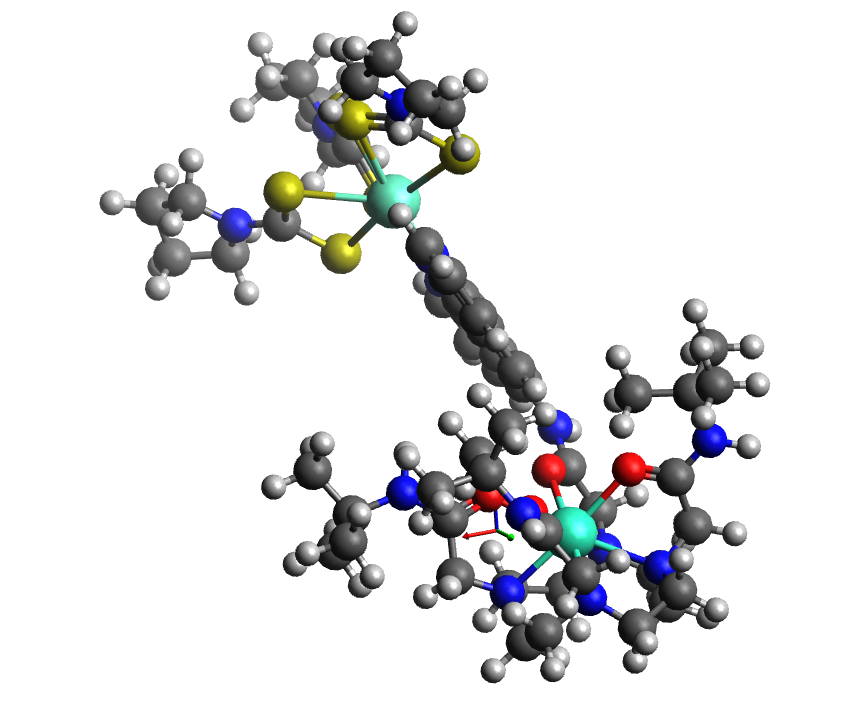


Fig. S13. Optimized structure of **cycTb-phEu** in MOPAC using RM1.

**NMR, MS and HPLC results**

The detail of characterization of intermediates, ligand and complexes is shown in Figs. S14 – S37.

Figure S14. ^1^H NMR spectrum of compound **1**.

Figure S15. ^13^C NMR spectrum of compound **1**.


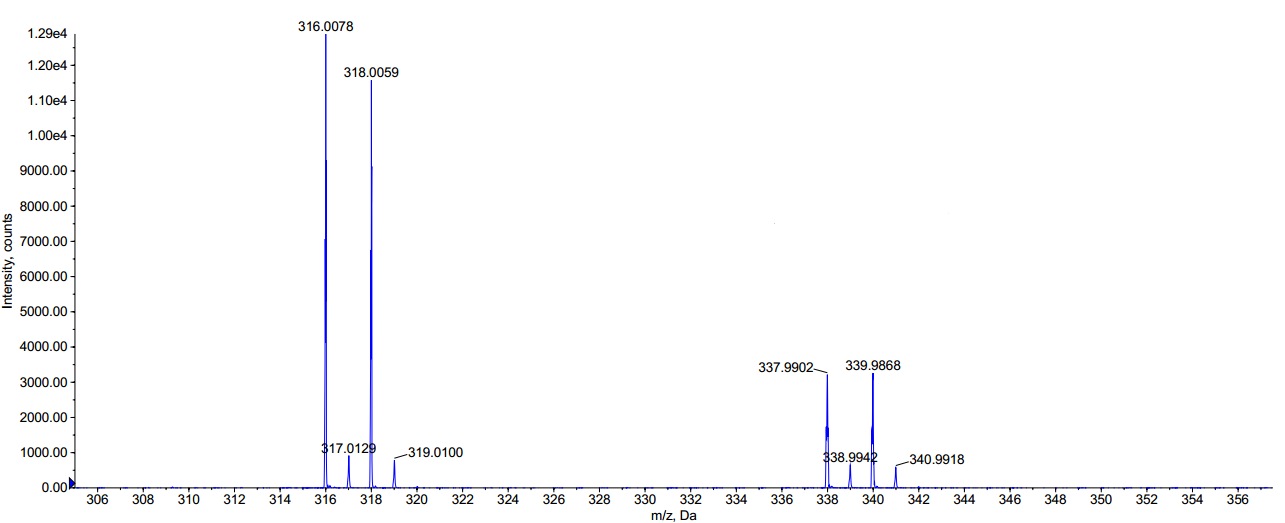


Figure S16. MS of compound **1**.

Figure S17. ^1^H NMR Spectrum of compound **2**.

Figure S18. ^13^C NMR spectrum of compound **2**.


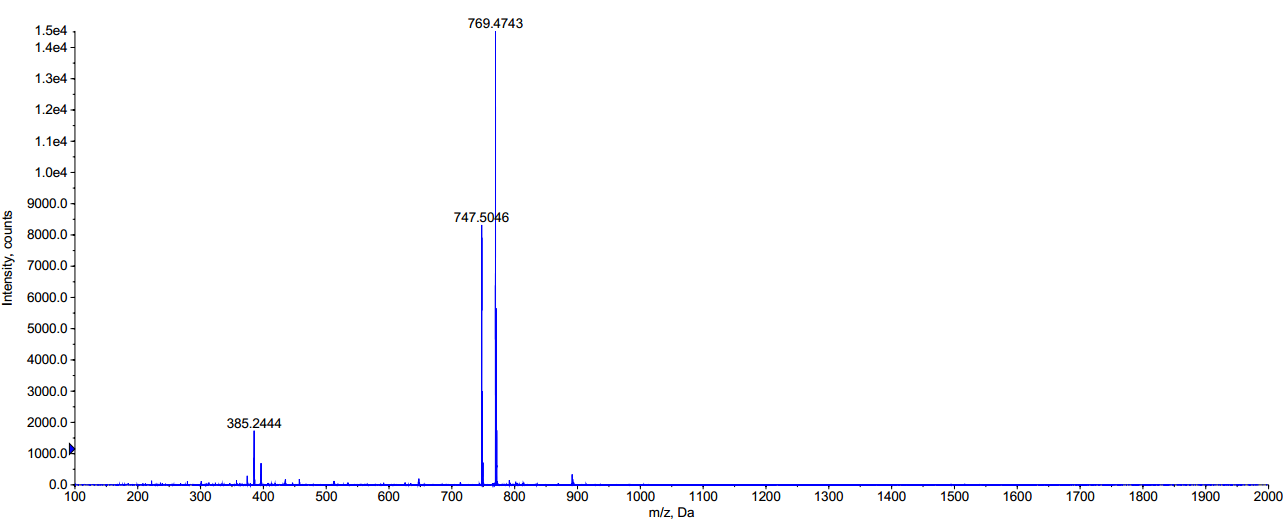


Figure S19. MS of compound **2**.

Figure S20. ^1^H NMR spectrum of **3a**.


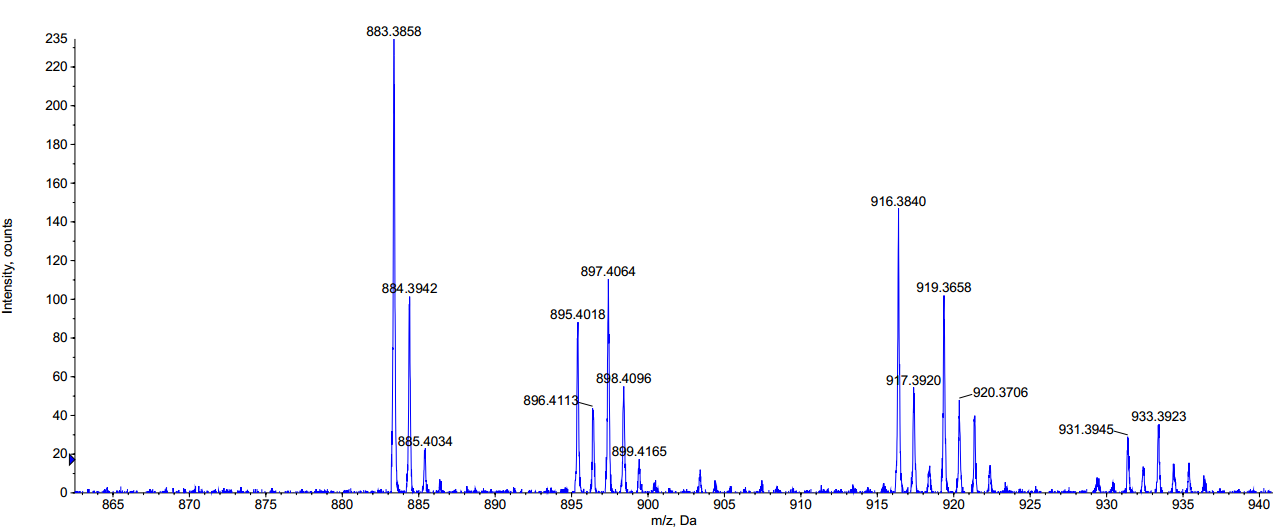


Figure S21. MS of **3a**


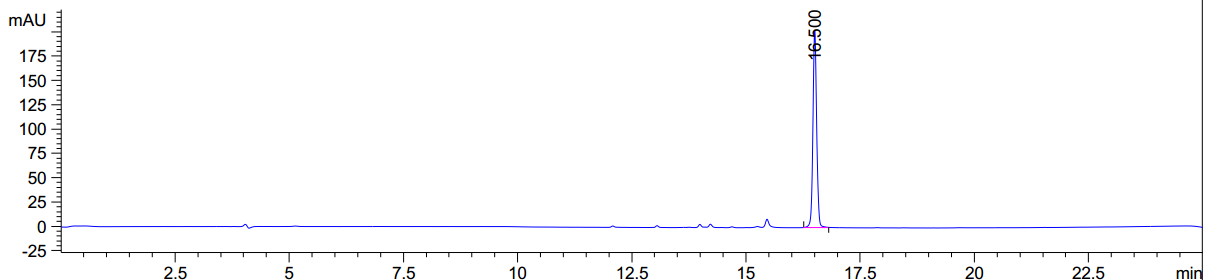


Figure S22. HPLC chromatogram of **3a**.


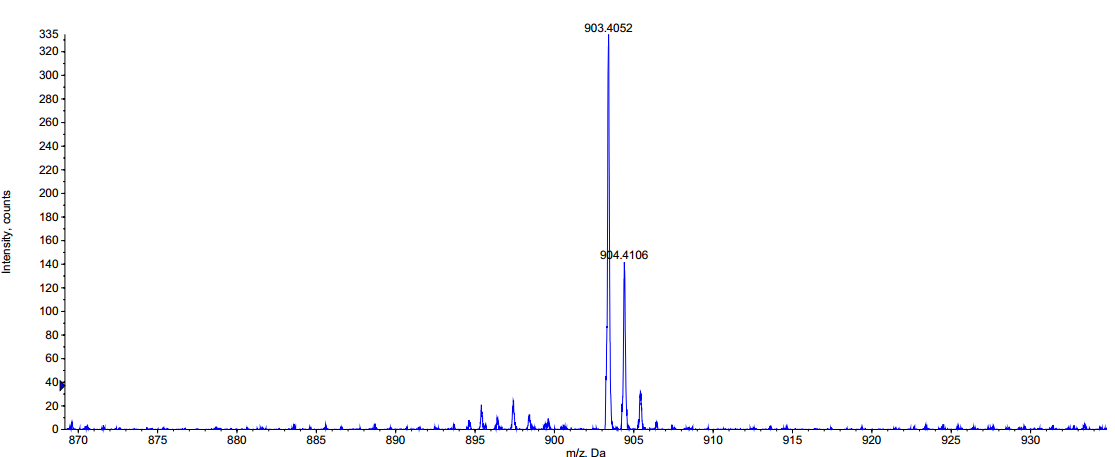


Figure S23. MS of **3b**.


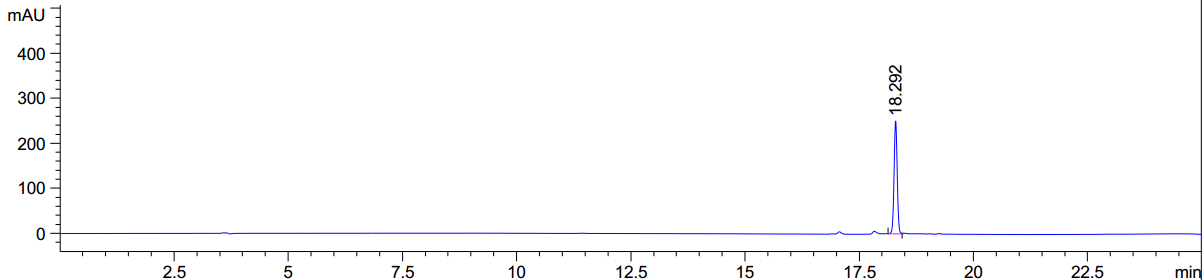


Figure S24. HPLC chromatogram of **3b**.


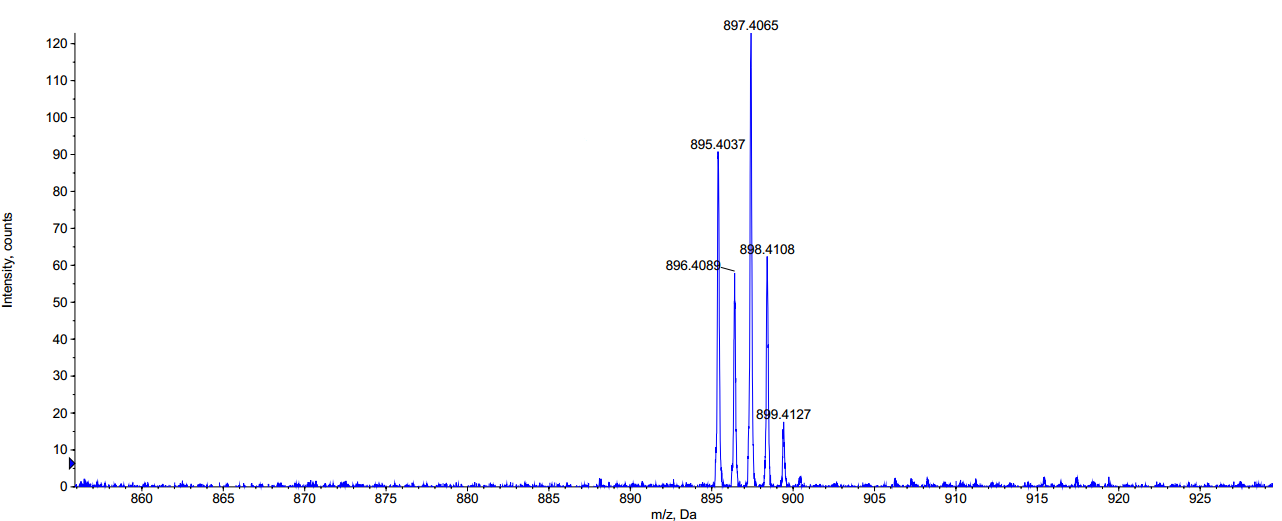


Figure S25. MS of **3c**.


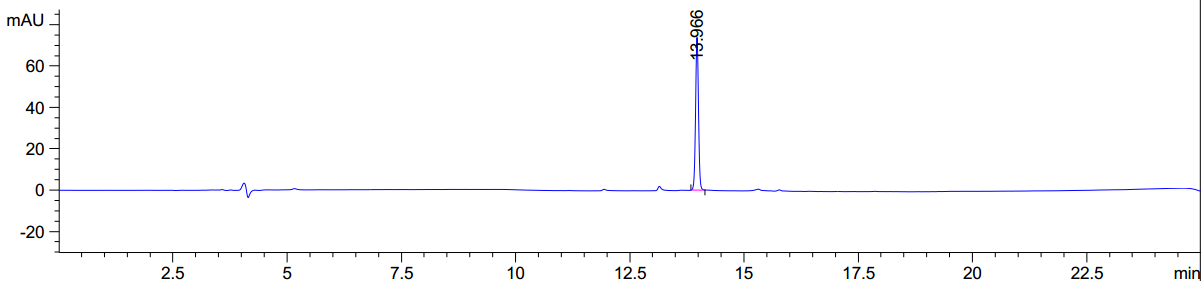


Figure S26. HPLC chromatogram of **3c**.

Figure S27. ^1^H NMR spectrum of **4a**


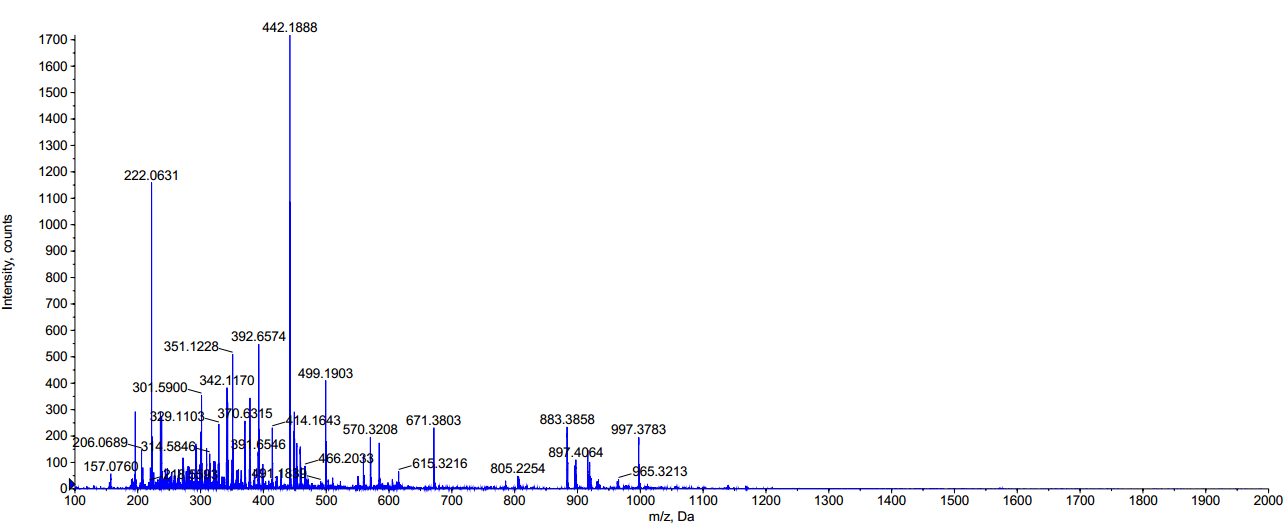


Figure S28. MS of **4a**.


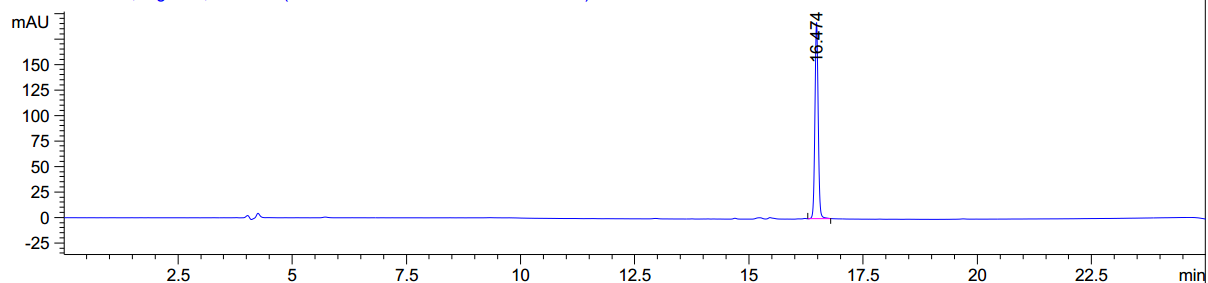


Figure S29. HPLC chromatogram of **4a**.


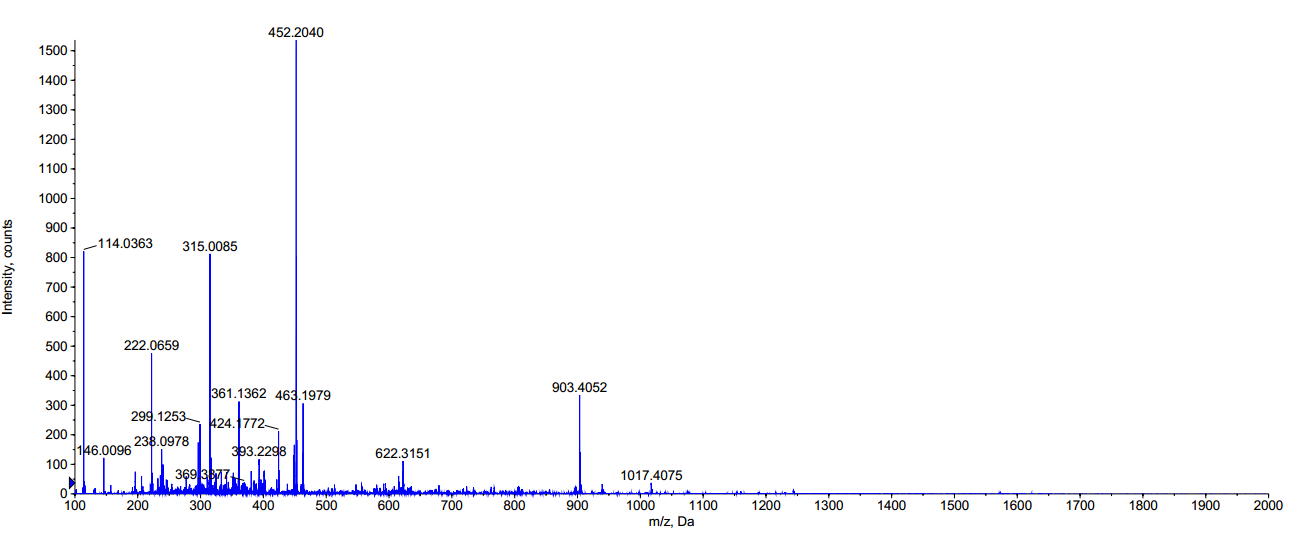


Figure S30. MS of **4b**.


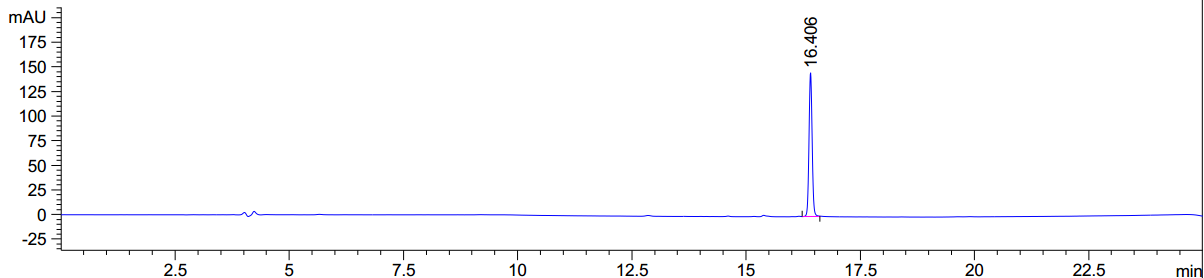


Figure S31. HPLC chromatogram of **4b**.


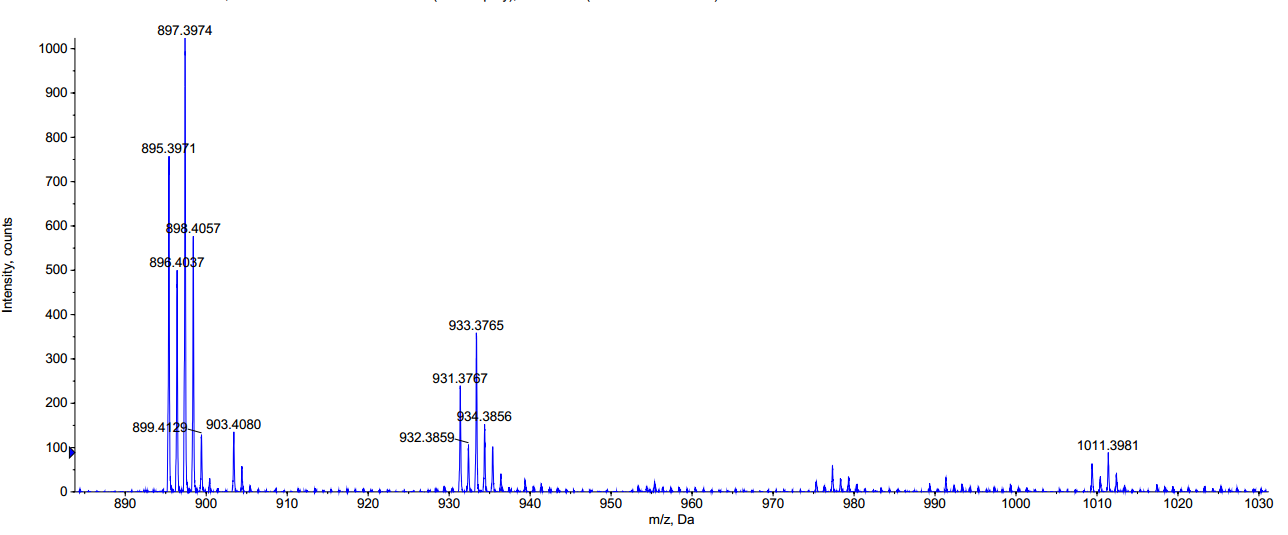


Figure S32. MS of **4c**.


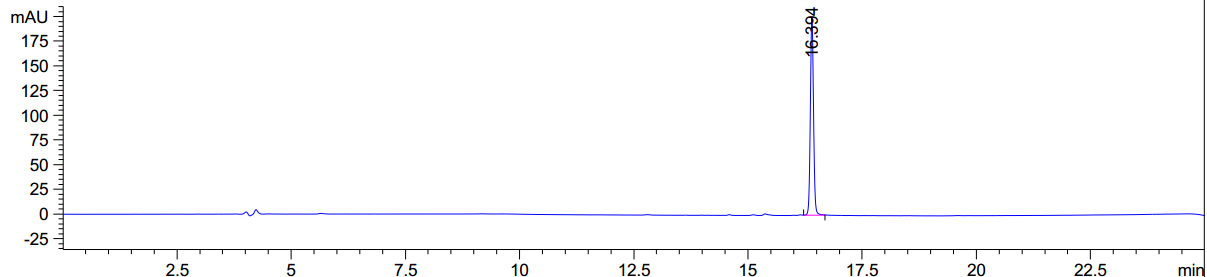


Figure S33. HPLC chromatogram of **4c**.

Figure S34. ^1^H NMR spectrum of **4d**.


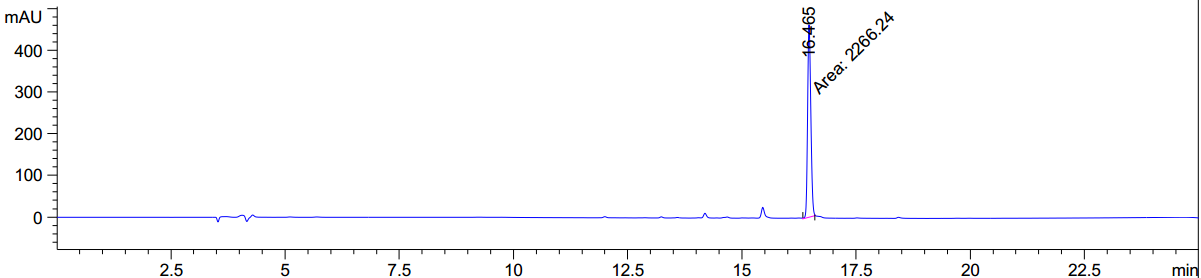


Figure S35. HPLC chromatogram of **4d**.


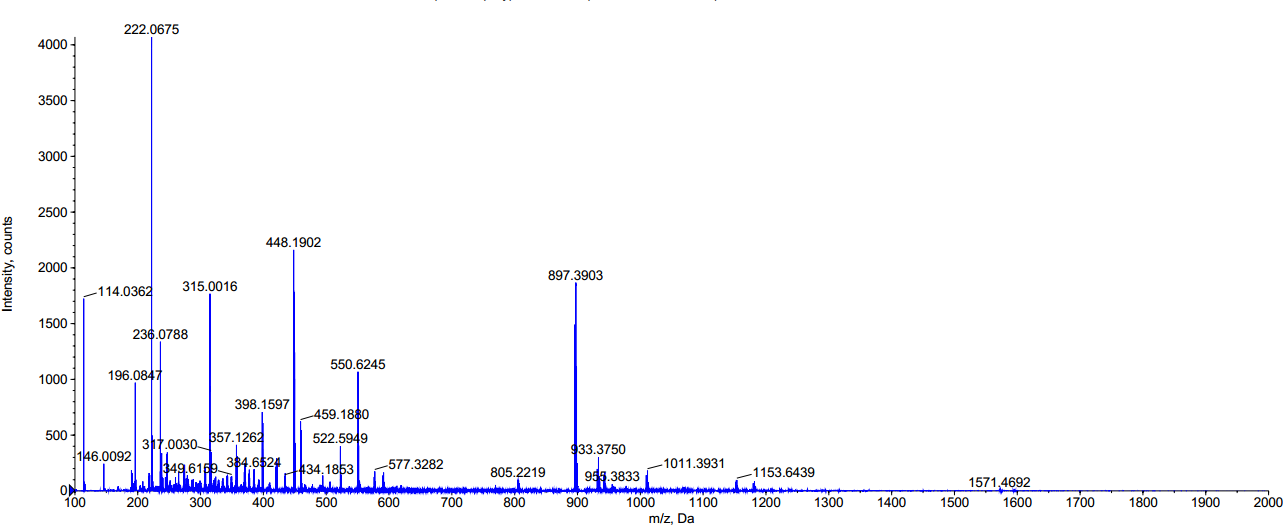


Figure S36. MS of **4e**.


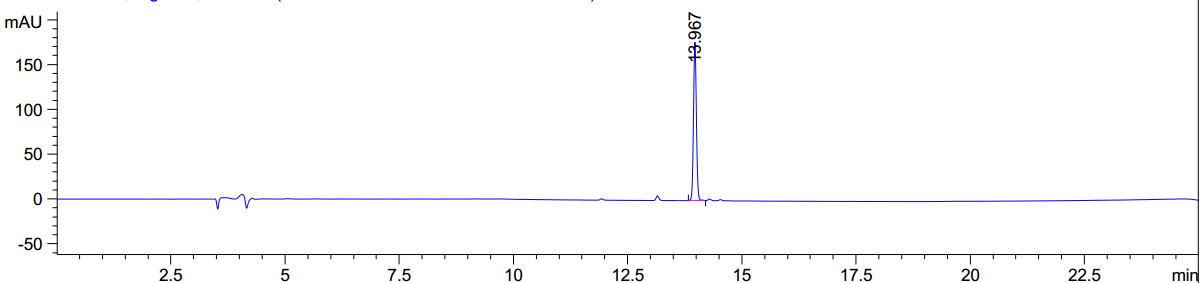


Figure S37. HPLC chromatogram of **4e**.

Table S14. The gradient of solvent for HPLC chromatogram. Column: Vision HT C18 HL 5 u, length 250 mm, Serial No. 5151920 ID 4.6 mm.

| Time (min) | 0.05% TFA in water (%) | ACN (%) |
| --- | --- | --- |
| 0 | 90 | 10 |
| 5 | 90 | 10 |
| 15 | 60 | 40 |
| 20 | 90 | 10 |
| 25 | 0 | 100 |

**References**

1. Miyata, K. *et al.* Chameleon luminophore for sensing temperatures: control of metal-to-metal and energy back transfer in lanthanide coordination polymers. *Angew. Chem. Int. Ed.* **52**, 6413-6416 (2013).

2. Li, J. H. *et al.* Luminescence properties and energy transfer of YGa_1.5_Al_1.5_(BO_3_)_4_:Tb^3+^,Eu^3+^ as a multi-colour emitting phosphor for WLEDs. *J. Mater. Chem. C* **5**, 6294-6299 (2017).

3. Bettinelli, M., Flint, C. D. Non-resonant energy transfer between Tb^3+^ and Eu^3+^ in the cubic hexachloroelpasolite crystals Cs_2_NaTb_1-x_Eu_x_Cl_6_ (x=0.01-0.15). *J. Phys-condens. Mat*. **2**, 8417-8426 (1990).

4. Lill, D. T., Bettencourt-Dias, A., Cahill, C. L. Exploring lanthanide luminescence in metal-organic frameworks:  synthesis, structure, and guest-sensitized luminescence of a mixed europium/terbium-adipate framework and a terbium-adipate framework. *Inorg. Chem.* **46**, 3960-3965 (2007).

5. Berry, M. T., May, P. S., Hu, Q. Calculated and observed Tb^3+^(^5^D_4_) → Eu^3+^ electronic energy transfer rates in Na_3_[Tb_0.01_Eu_0.99_(oxydiacetate)_3_]-2NaClO_4_-6H_2_O. *J. Lumin.* **71**, 269-283 (1997).

6. Bettinelli, M., Piccinelli, F., Speghini, A., Ueda, J., Tanabe, S. Excited state dynamics and energy transfer rates in Sr_3_Tb_0.90_Eu_0.10_(PO_4_)_3_. *J. Lumin*. **132**, 27-29 (2012).

7. Carrasco, I., Bartosiewicz, K., Nikl, M., Piccinelli, F., Bettinelli, M. Energy transfer processes in Ca_3_Tb_2−x_Eu_x_Si_3_O_12_ (x=0–2). *Opt. Mater.* **48**, 252-257 (2015).

8. Hatanaka, M. *et al.* Organic linkers control the thermosensitivity of the emission intensities from Tb(III) and Eu(III) in a chameleon polymer. *Chem. Sci.* **8**, 423-429 (2017).

9. Cadiau, A. *et al*. Ratiometric nanothermometer based on an emissive Ln^3+^-organic framework. *ACS Nano* **7**, 7213-7218 (2013).

10. Marciniak, L., Prorok, K., Francés-Soriano, L., Pérez-Prieto, J. & Bednarkiewicz, A. A broadening temperature sensitivity range with a core–shell YbEr@YbNd double ratiometric optical nanothermometer. *Nanoscale* **8**, 5037-5042 (2016).

11. Zhao, D. *et al.* Design and synthesis of an MOF thermometer with high sensitivity in the physiological temperature range. *Inorg. Chem.* **54**, 11193-11199 (2015).

12. Ananias, D., Almeida Paz, F. A., Carlos, L. D. & Rocha, J. Near-infrared ratiometric luminescent thermometer based on a new lanthanide silicate. *Chem. Eur. J.* **24**, 11926-11935 (2018).

13. Ximendes, E. C. *et al.* In Vivo Subcutaneous thermal video recording by supersensitive infrared nanothermometers. *Adv. Funct. Mater.* **27**, 1702249 (2017).

14. Brites, C. D. S. *et al.* Ratiometric highly sensitive luminescent nanothermometers working in the room temperature range. Applications to heat propagation in nanofluids. *Nanoscale* **5**, 7572-7580 (2013).

15. Ximendes, E. C. *et al.* Unveiling in vivo subcutaneous thermal dynamics by infrared luminescent nanothermometers. *Nano lett.* **16**, 1695-1703 (2016).

16. Avogadro: an open-source molecular builder and visualization tool. *Version 1.1.1.* [*http://avogadro.cc/*](http://avogadro.cc/).

17. Allouche A. R. Gabedit, Version 2.1.0, Laboratoire de spectrométrie ionique et moléculaire - UMR 5579 CNRS et Université Claude Bernard Lyon1.

18. Stewart J. J. P. MOPAC 2016 (Molecular Orbital PACkage), MOPAC2016, Stewart Computational Chemistry, Colorado Springs, CO, USA, HTTP://OpenMOPAC.net (2016).

19. Filho, M. A., Dutra, J. D., Rocha, G. B., Simas, A. M. & Freire, R. O. Parameters for the RM1 quantum chemical calculation of complexes of the trications of thulium, ytterbium and lutetium. *PLoS One* **11**, 1-11 (2016).

20. Dutra, J. D., Bispo, T. D., Freire, R. O. LUMPAC lanthanide luminescence software: Efficient and user friendly. *J. Comput. Chem.* **35**, 772-775 (2014).

21. Neese, F. The ORCA program system. *Wiley Interdiscip. Rev.: Comput. Mol. Sci.* **2**, 73-78 (2012).

22. Grimme, S., Brandenburg, J. G., Bannwarth, C. & Hansen, A. Consistent structures and interactions by density functional theory with small atomic orbital basis sets. *J. Chem. Phys.* **143**, 054107 (2015).

23. Bao G. C. *et al.* Reversible and sensitive Hg^2+^ detection by a cell-permeable ytterbium complex. *Inorg. Chem.* **57**, 120-128 (2018).

24. Yu, Y. L. *et al.* Self-calibrating optic thermometer based on dual-emission nanocomposite. *J. Alloy. and Compd.* **730**, 12-16 (2018).
